# Supplementary material for: Can the fusion of motion capture and 3D medical imaging reduce the extrinsic variability due to marker misplacements?
Source: PLoS One. 2020 Jan 29;15(1):e0226648. doi: 10.1371/journal.pone.0226648 (PMC6988975; doi:10.1371/journal.pone.0226648)

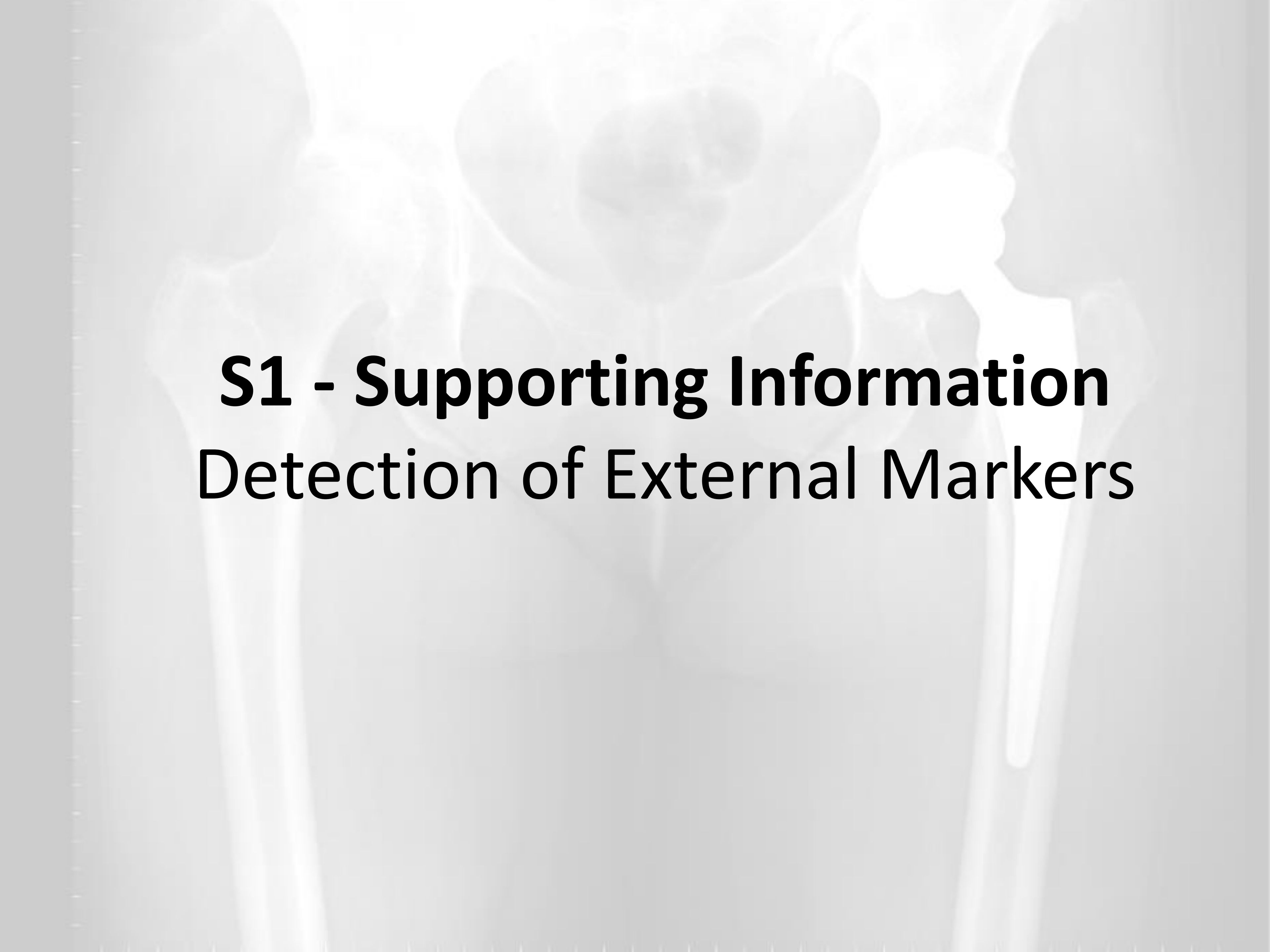

S1 - Supporting Information

Detection of External Markers

Supporting info to: Can the fusion of motion capture and 3D medical imaging reduce the extrinsic variability due to marker misplacements?
X. Gasparutto, J. Wegrzyk, K. Rose-Dulcina, D. Hannouche, S. Armand

External Markers Detection

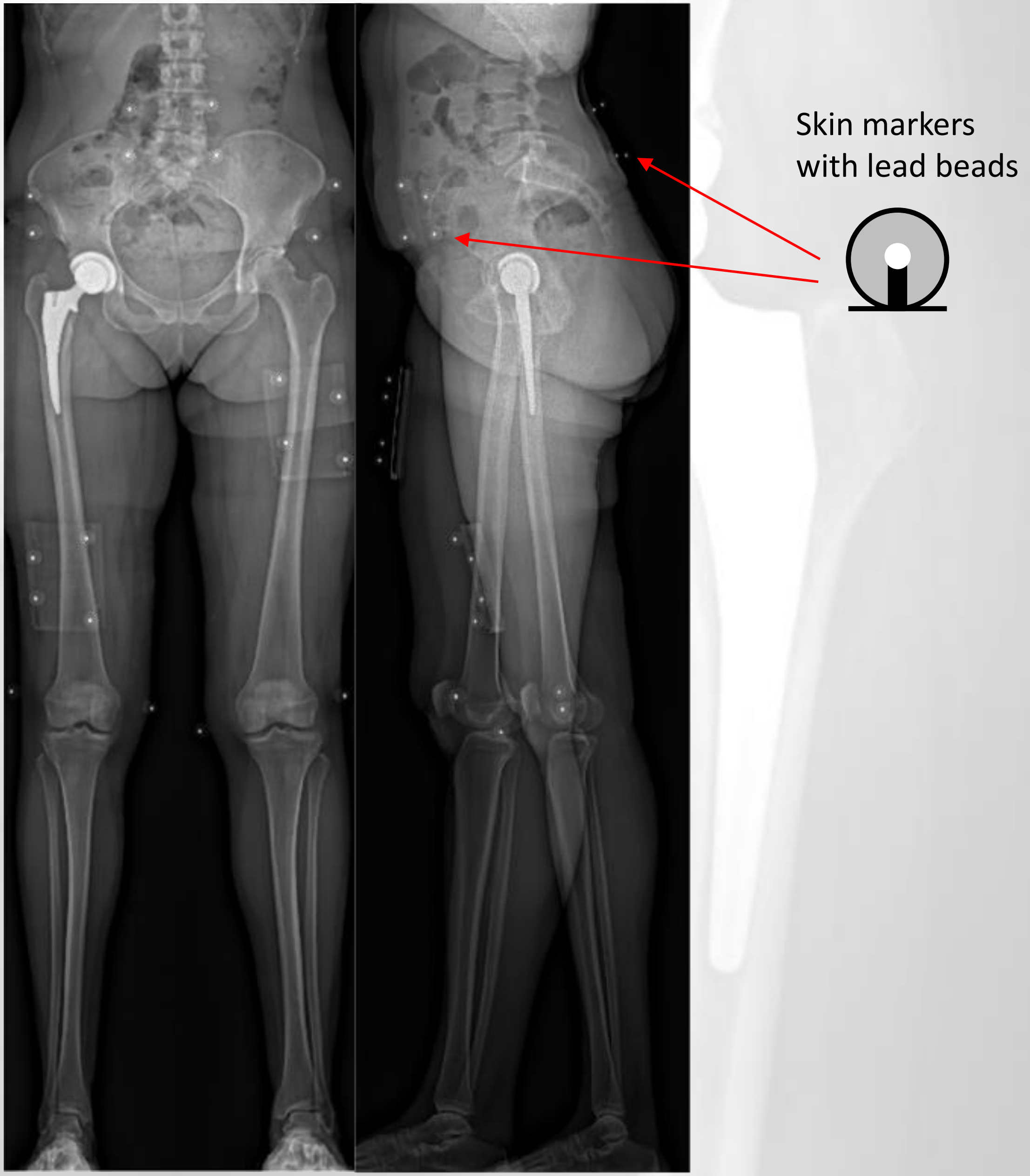

External Markers Detection

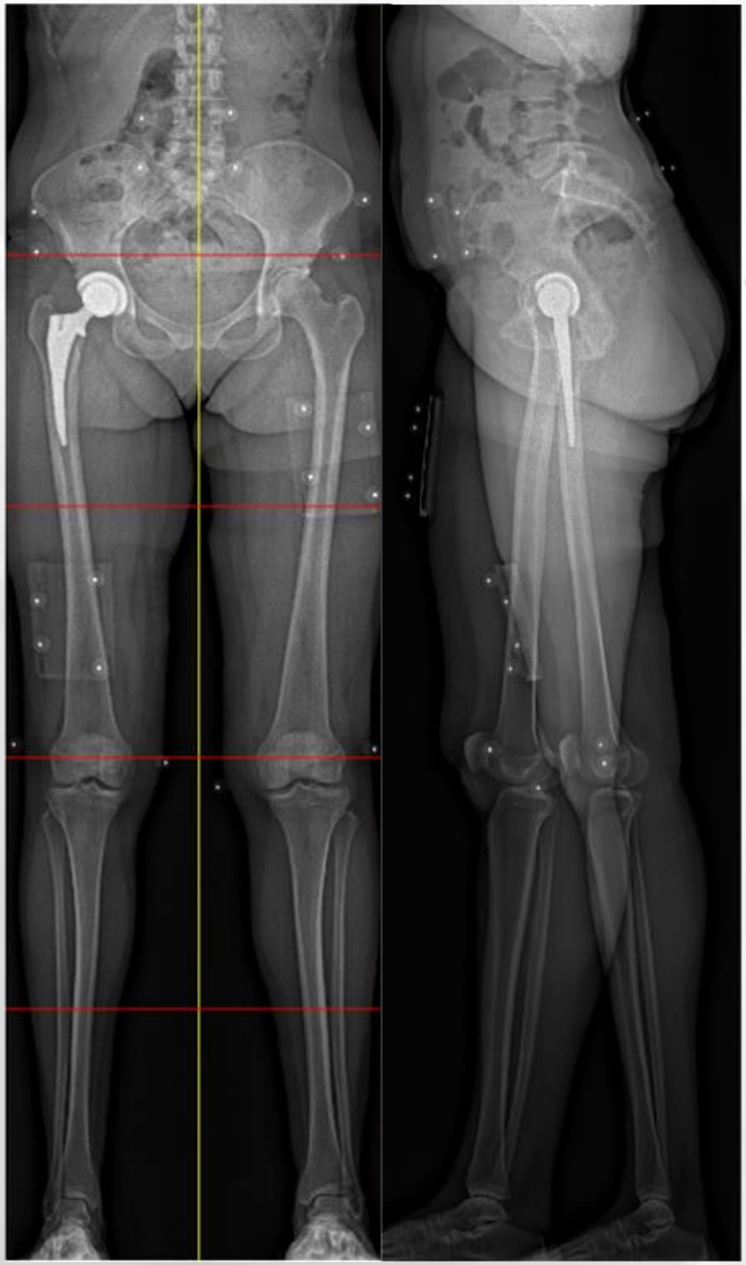

Step 1: Threshold on moving window
-> everything below a percentage of max luminosity in the window is set to 0
-> Threshold is between 60% and 90%

External Markers Detection

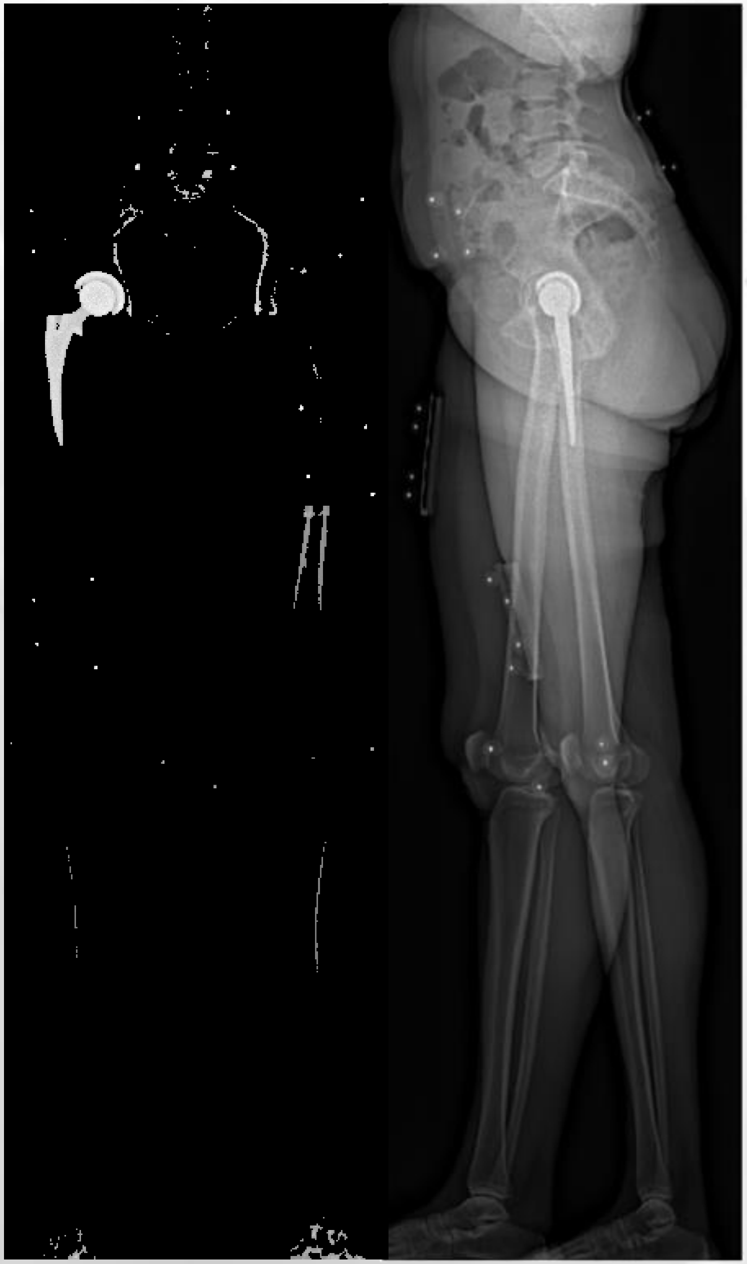

Step 2: Filters (Matlab functions)
- bwareaopen: remove smallest elements
- imfill: fill image regions and holes

External Markers Detection

Step 3: Identify points

- regionprops: get region properties

- Constraints on ratio Minor/Major axis
  - Ratio should be > 0.8
  - Circle would be 1

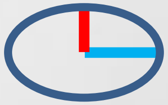

- Constraint on minimal size (8px)

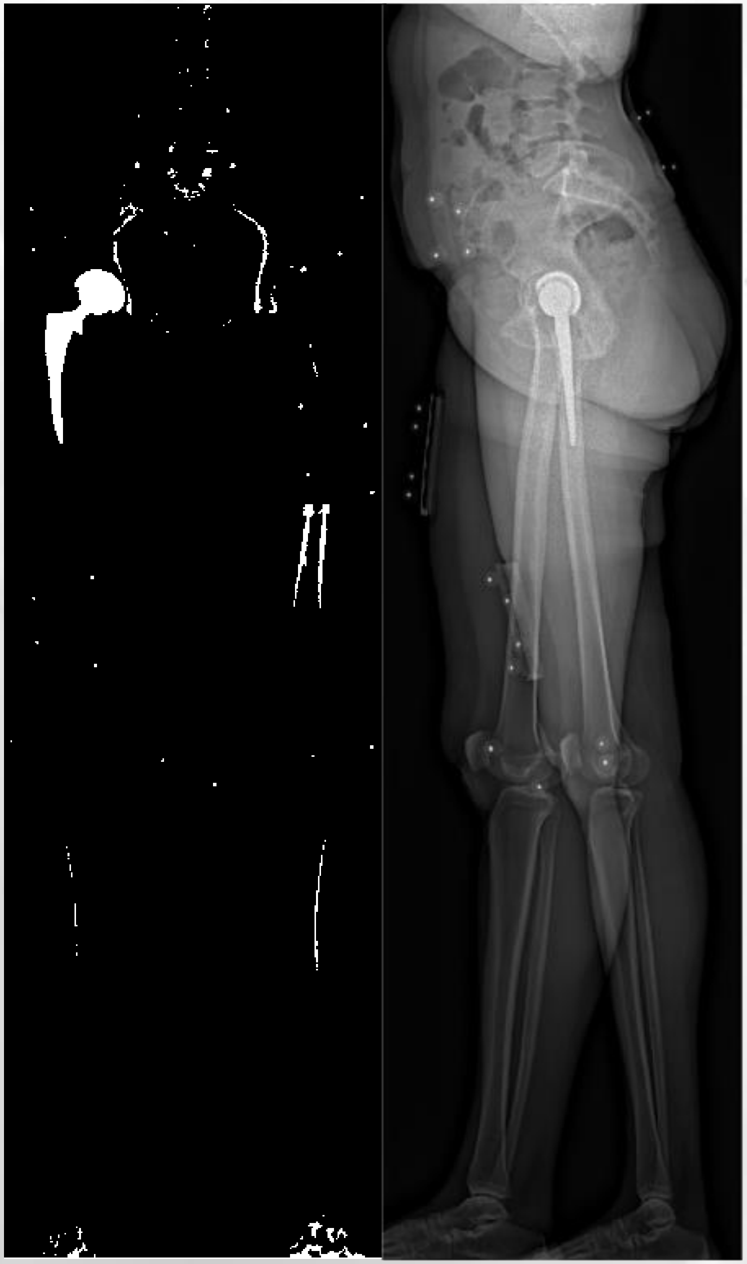

External Markers Detection

Step 3: Identify points

- regionprops: get region properties

- Constraints on ratio Minor/Major axis
    - Ratio should be > 0.8
    - Circle would be 1

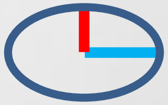

- Constraint on minimal size (8px)

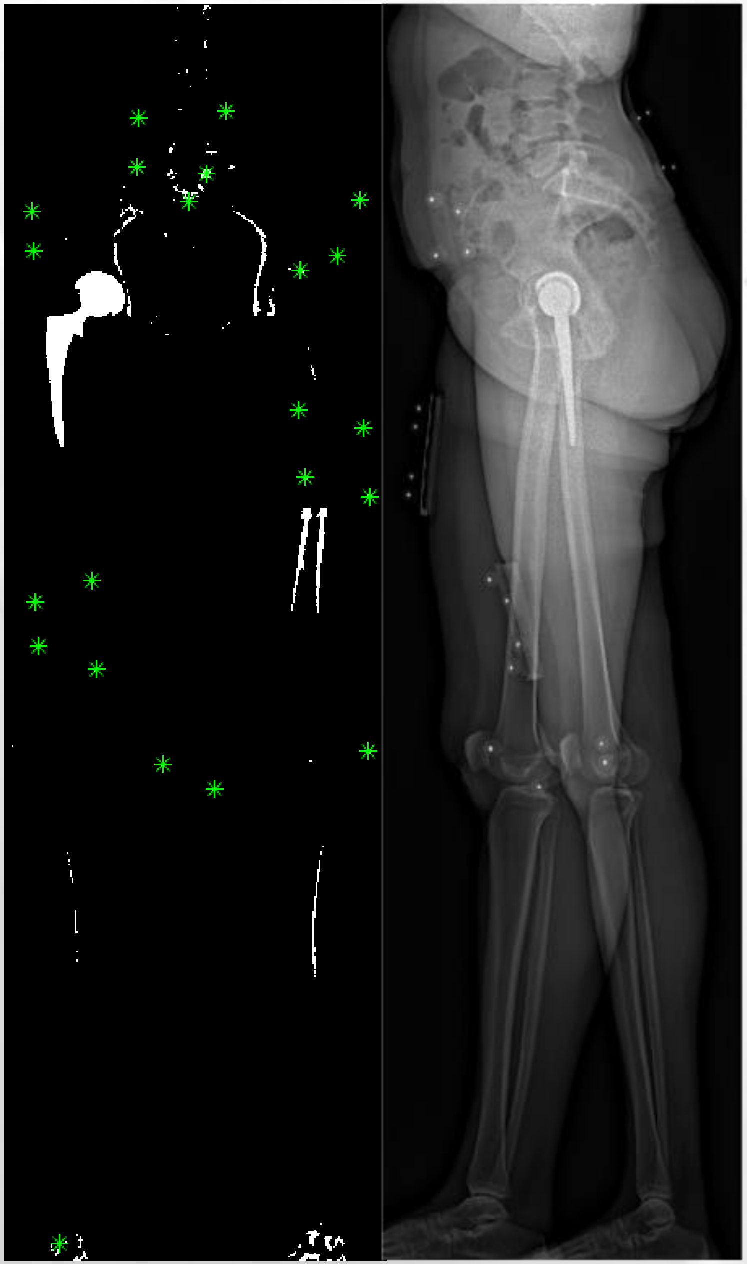

External Markers Detection

Step 4: Points on sagittal view
- Take strip of image at marker height
- Marker detection algorithm

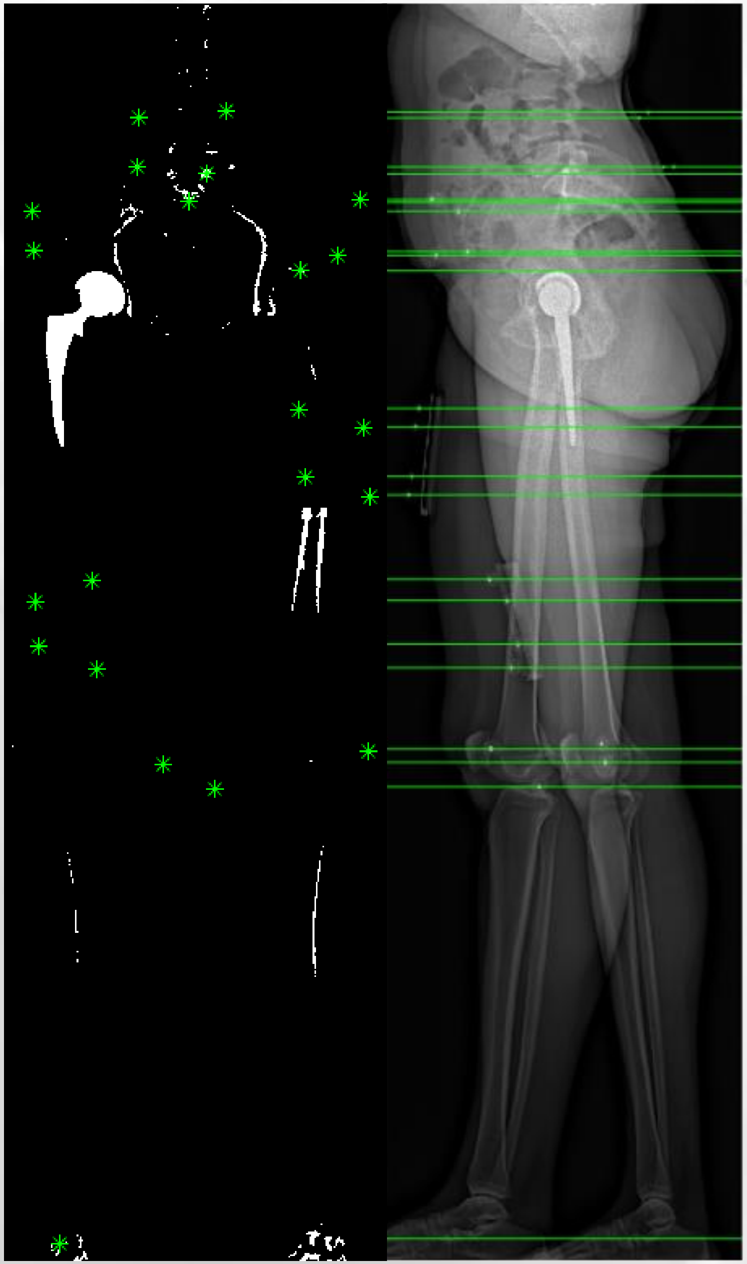

External Markers Detection

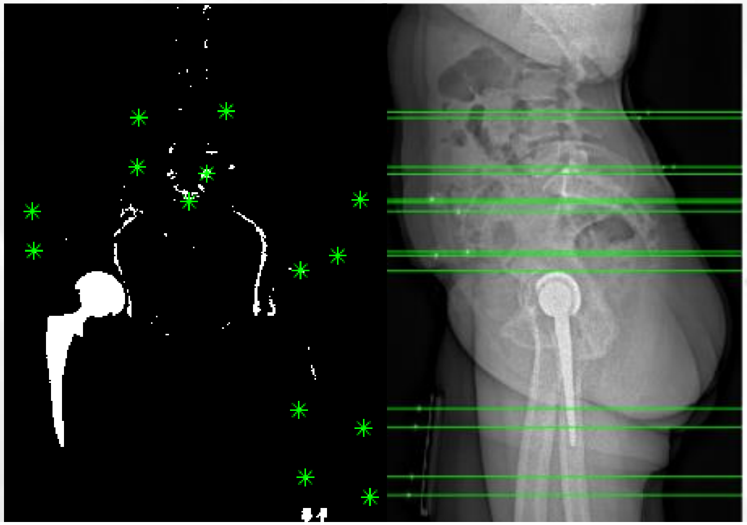

Step 4: Points on sagittal view

- Take strip [illegible]
- Marker de[illegible]

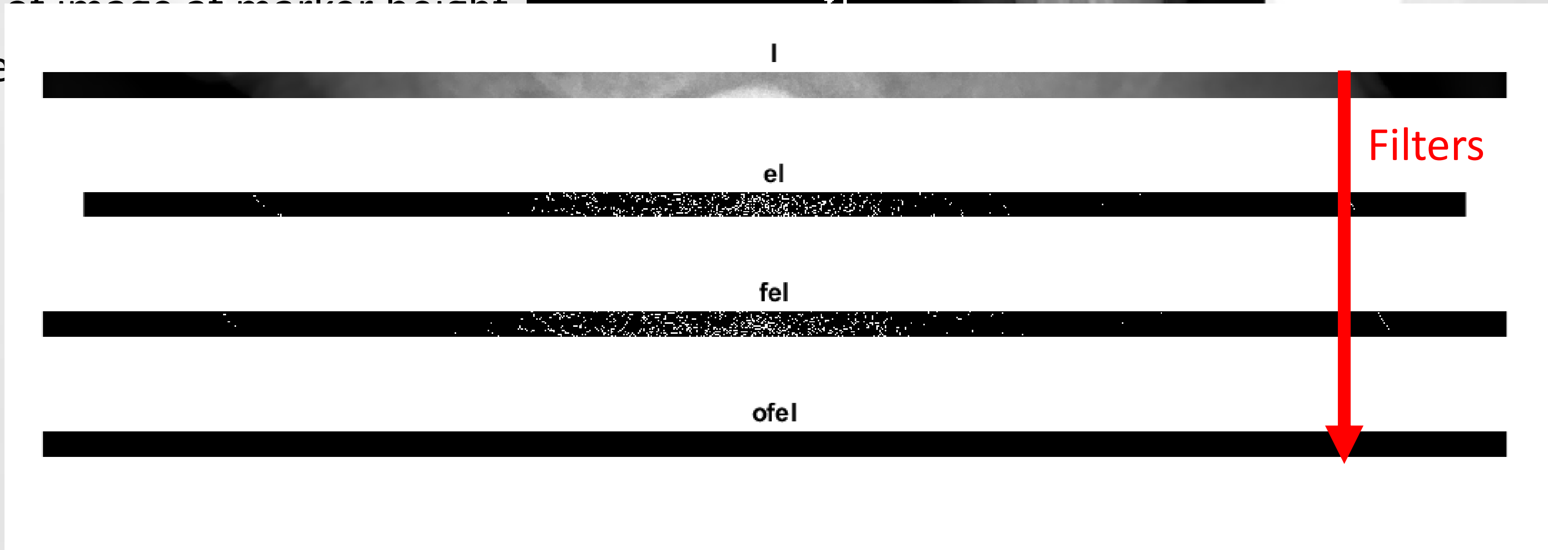

No point on sagittal view

-> Point is removed

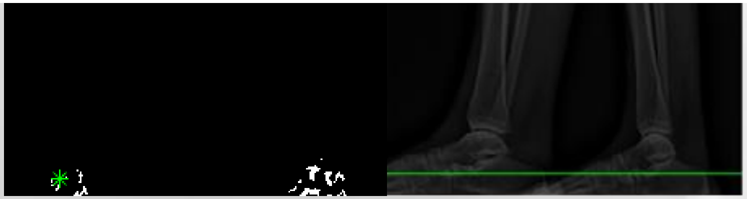

Supporting info to: Can the fusion of motion capture and 3D medical imaging reduce the extrinsic variability due to marker misplacements?
X. Gasparutto, J. Wegrzyk, K. Rose-Dulcina, D. Hannouche, S. Armand

External Markers Detection

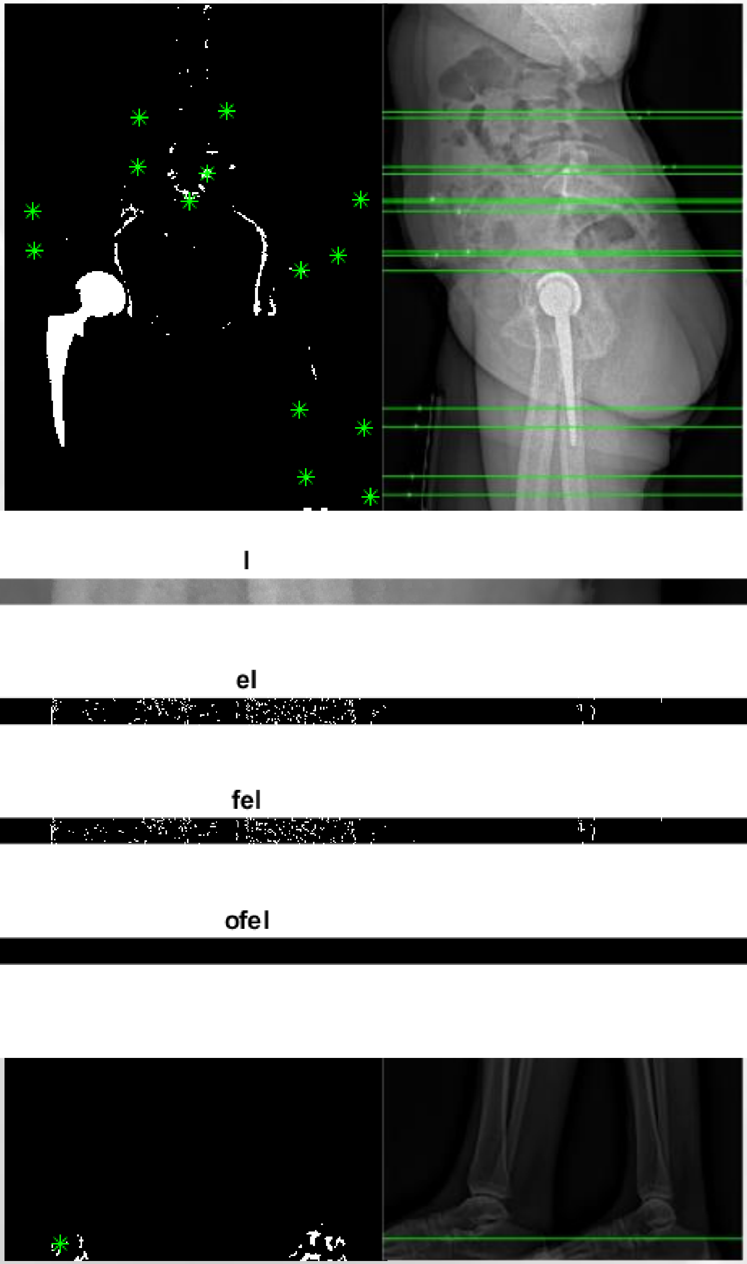

Step 4: Points on sagittal view

- Take stri[illegible]
- Marker [illegible]

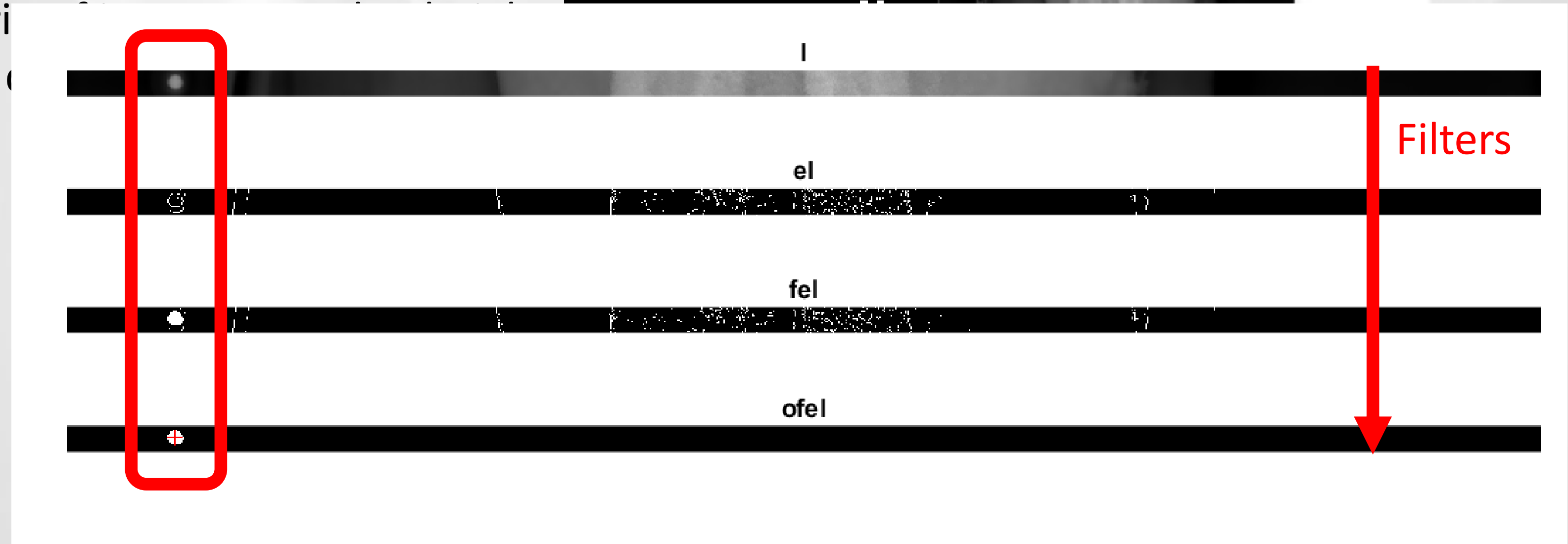

Point on sagittal view
-> Point is a marker

External Markers Detection

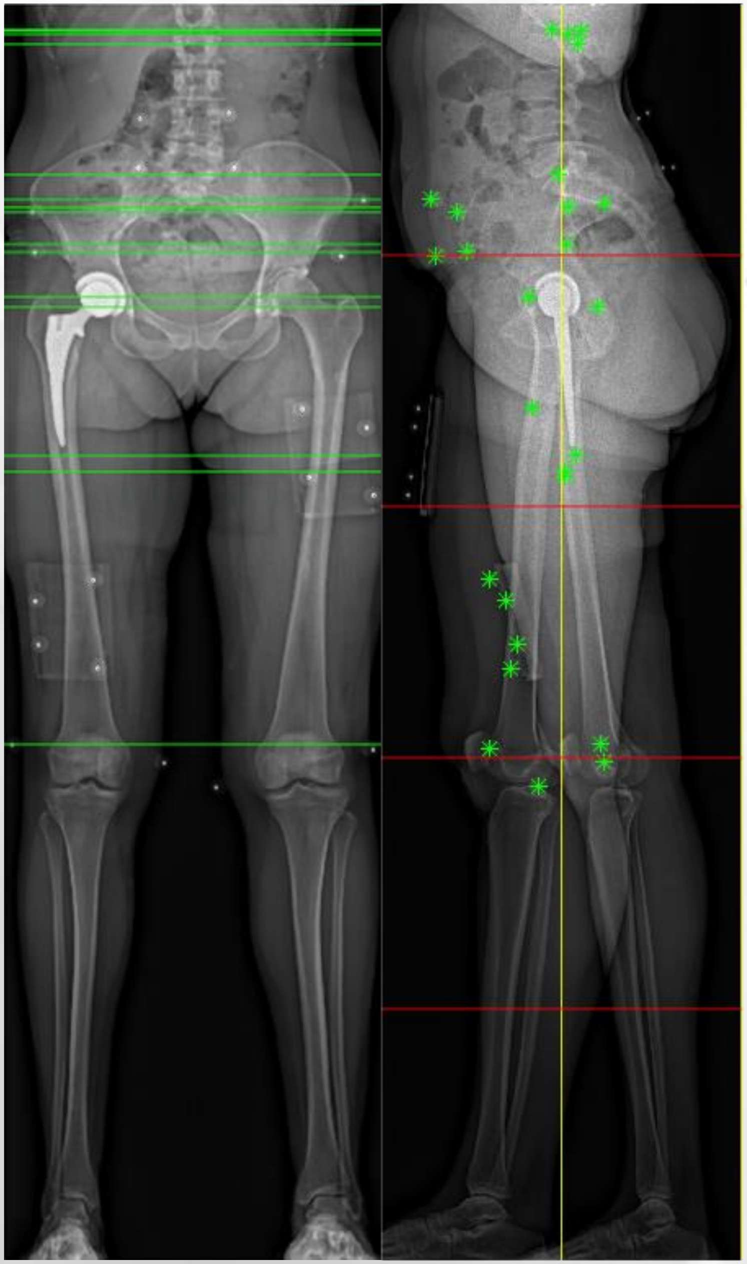

Step 5 : Same but Sagit. to Frontal
- On sagittal: check if point is new
- On frontal: check if point is a marker
- If so save marker

X. Gasparutto, J. Wegrzyk, K. Rose-Dulcina, D. Hannouche, S. Armand

External Markers Detection

Step 6: Missing markers?
- Missing marker are identified manually
- Select zone manually
- Detection algorithm on zone

Step 7:
- Manual ID of Skin Markers

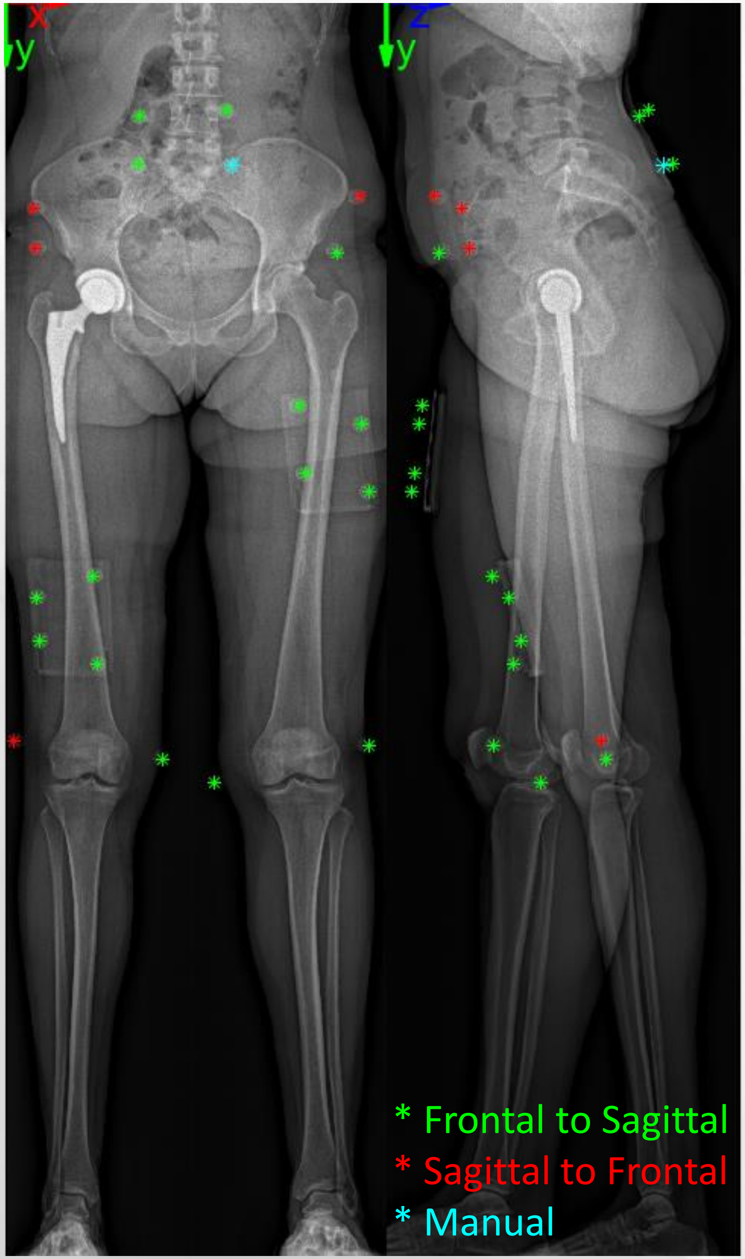

Supplement: S1 File — (PDF) [file pone.0226648.s001.pdf]
