# Supplementary material for: Can the fusion of motion capture and 3D medical imaging reduce the extrinsic variability due to marker misplacements?
Source: PLoS One. 2020 Jan 29;15(1):e0226648. doi: 10.1371/journal.pone.0226648 (PMC6988975; doi:10.1371/journal.pone.0226648)

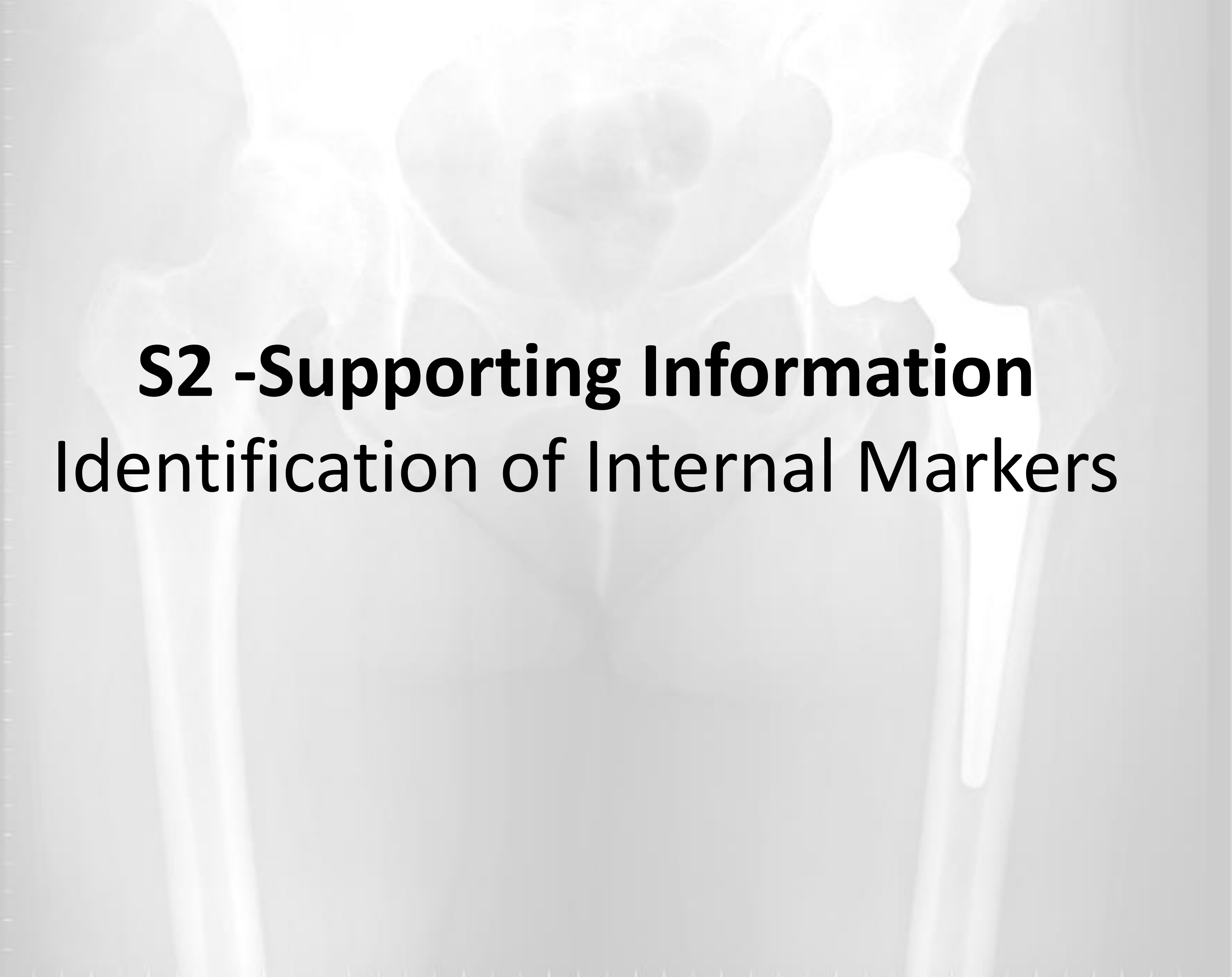

S2 -Supporting Information

Identification of Internal Markers

Hip Joint Centre

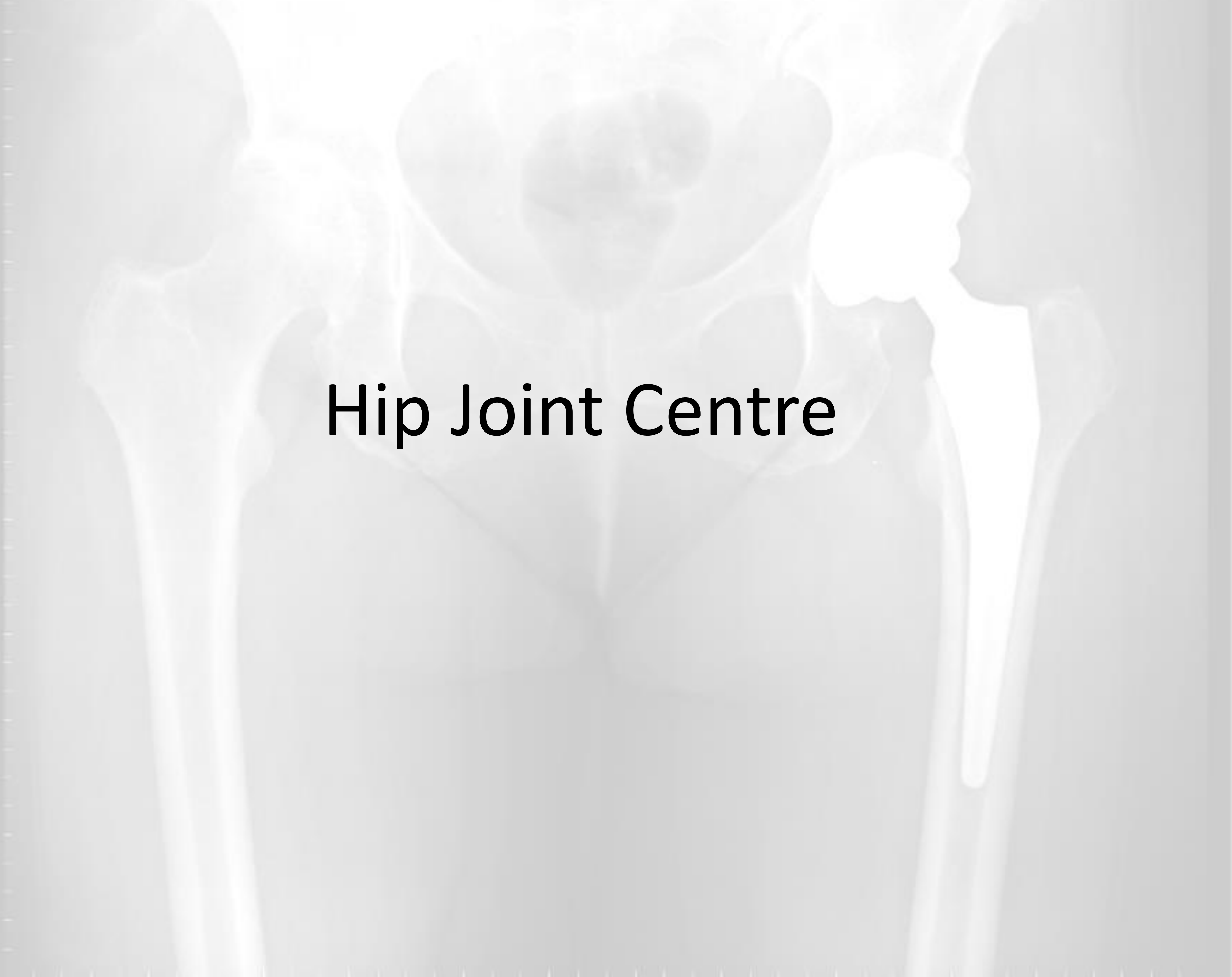

Hip Joint Center – step 1

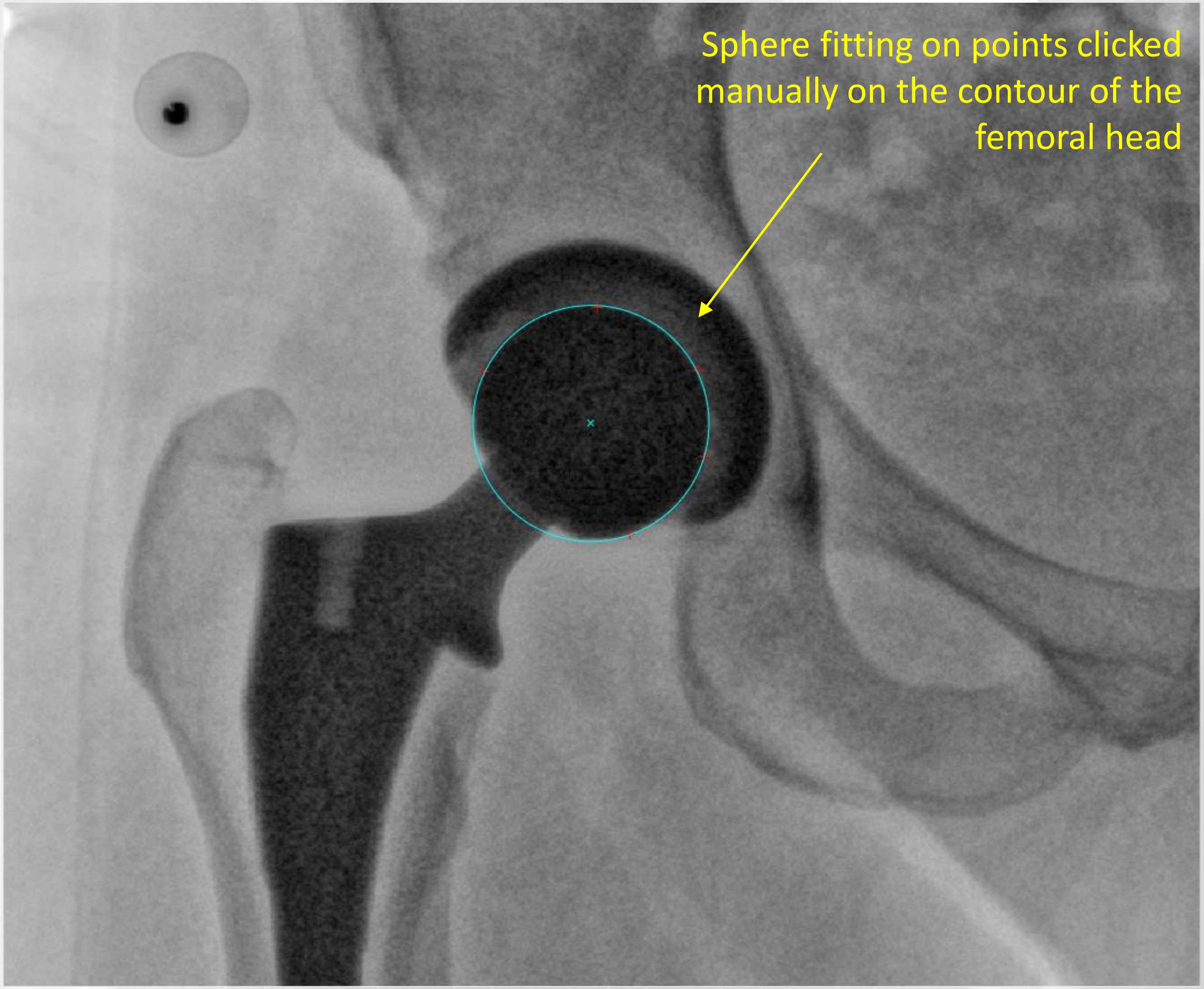

X. Gasparutto, J. Wegrzyk, K. Rose-Dulcina, D. Hannouche, S. Armand

Hip Joint Center – step 2

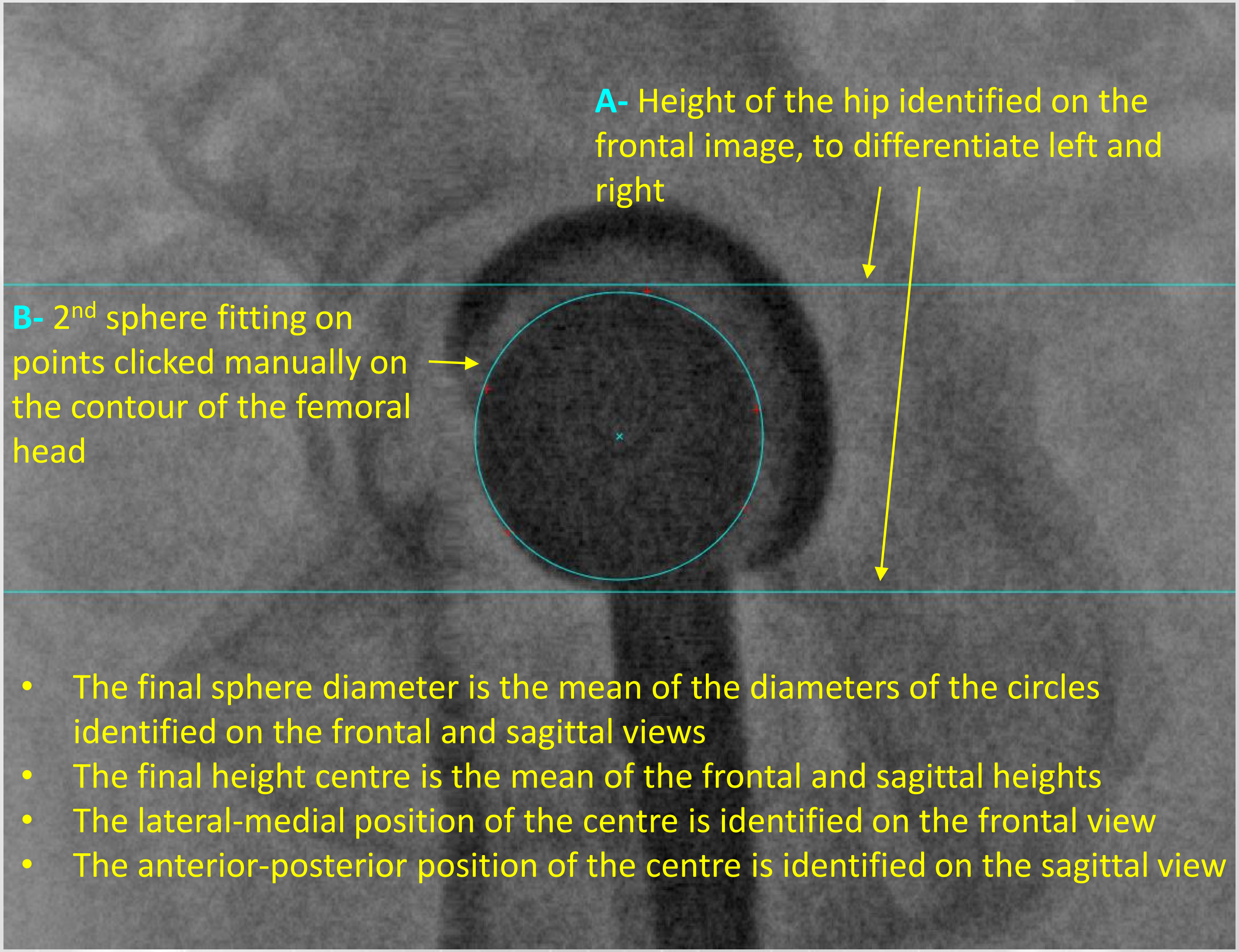

- The final sphere diameter is the mean of the diameters of the circles identified on the frontal and sagittal views
- The final height centre is the mean of the frontal and sagittal heights
- The lateral-medial position of the centre is identified on the frontal view
- The anterior-posterior position of the centre is identified on the sagittal view

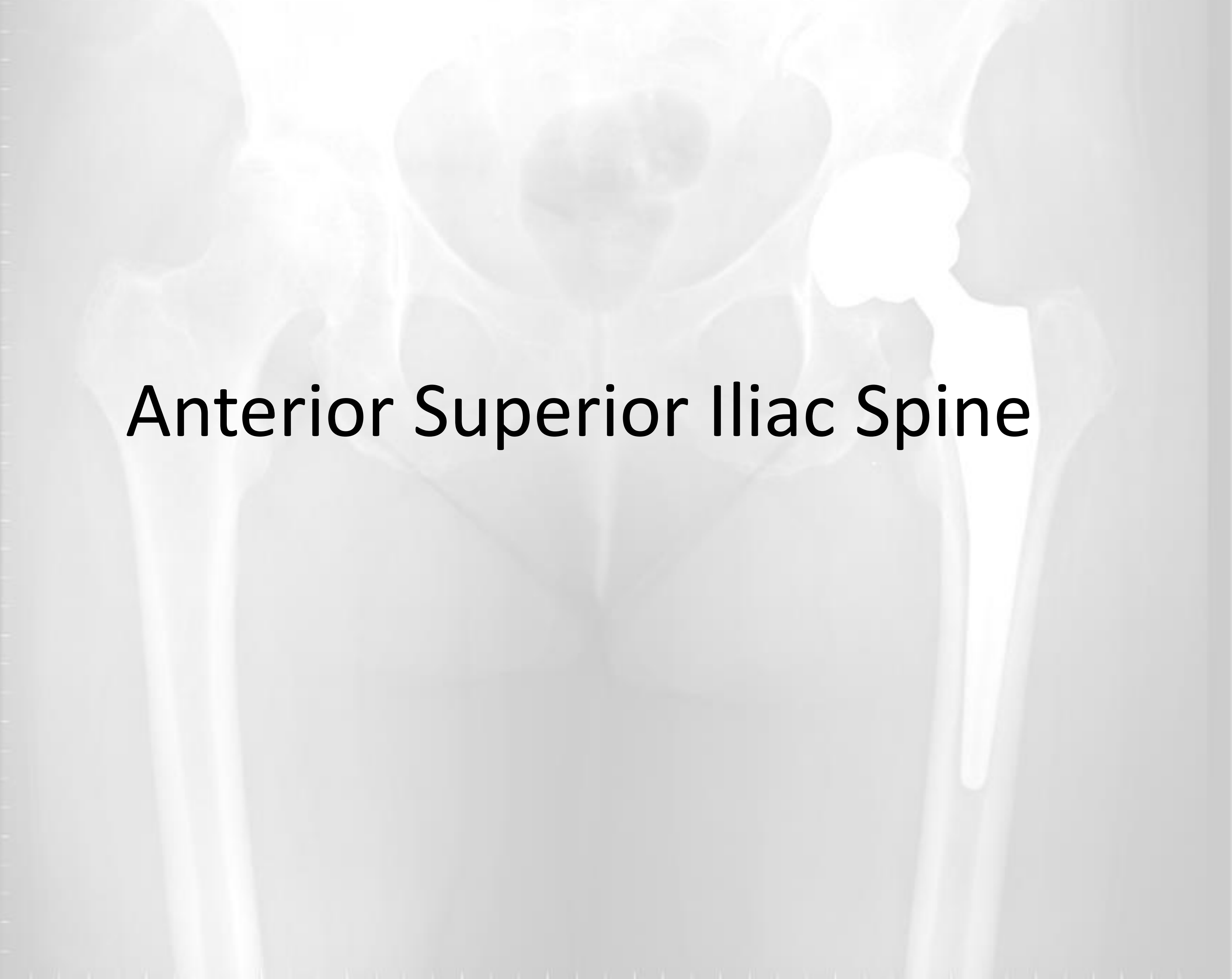

Anterior Superior Iliac Spine

Anterior superior iliac spine – Method 1 - Step 1

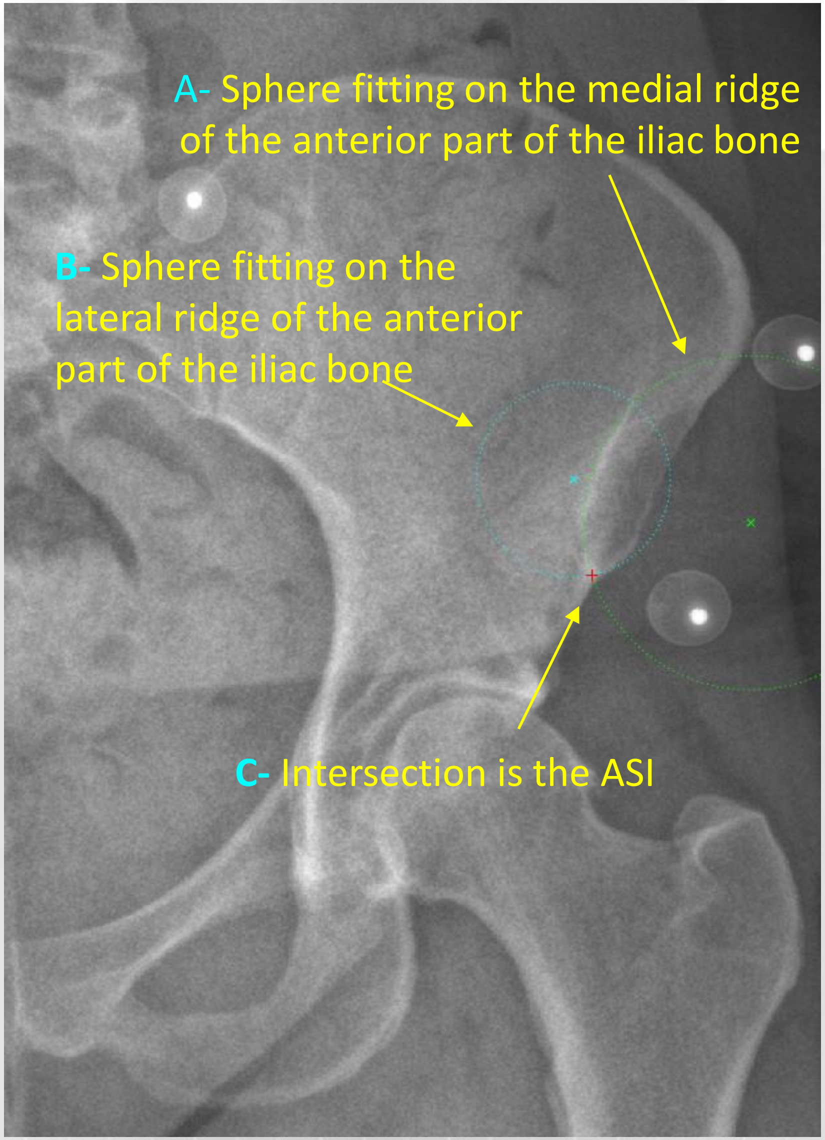

Anterior superior iliac spine – Method 1 - Step 2

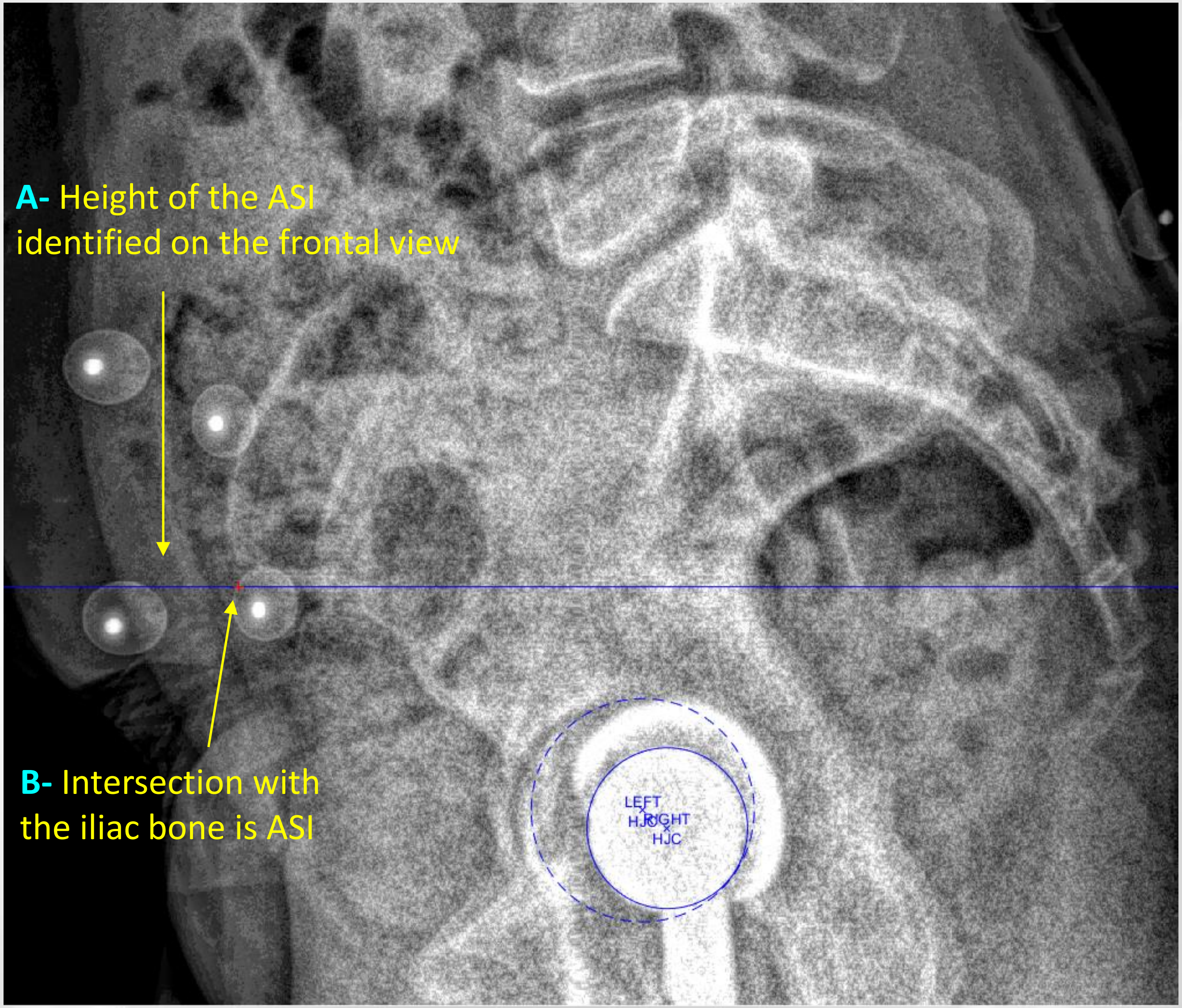

Anterior superior iliac spine – Method 2 - Step 1

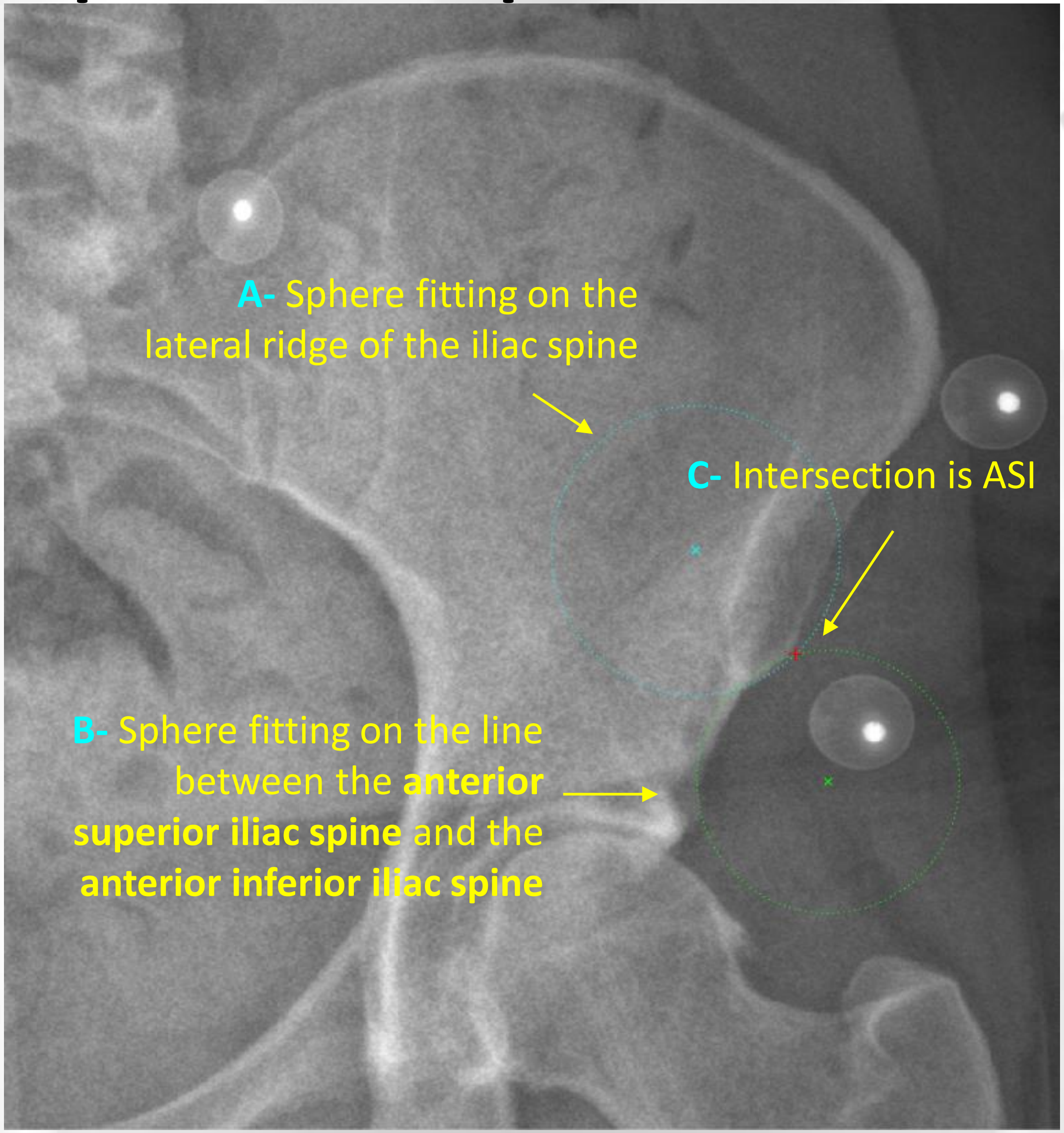

Anterior superior iliac spine – Method 1 - Step 2

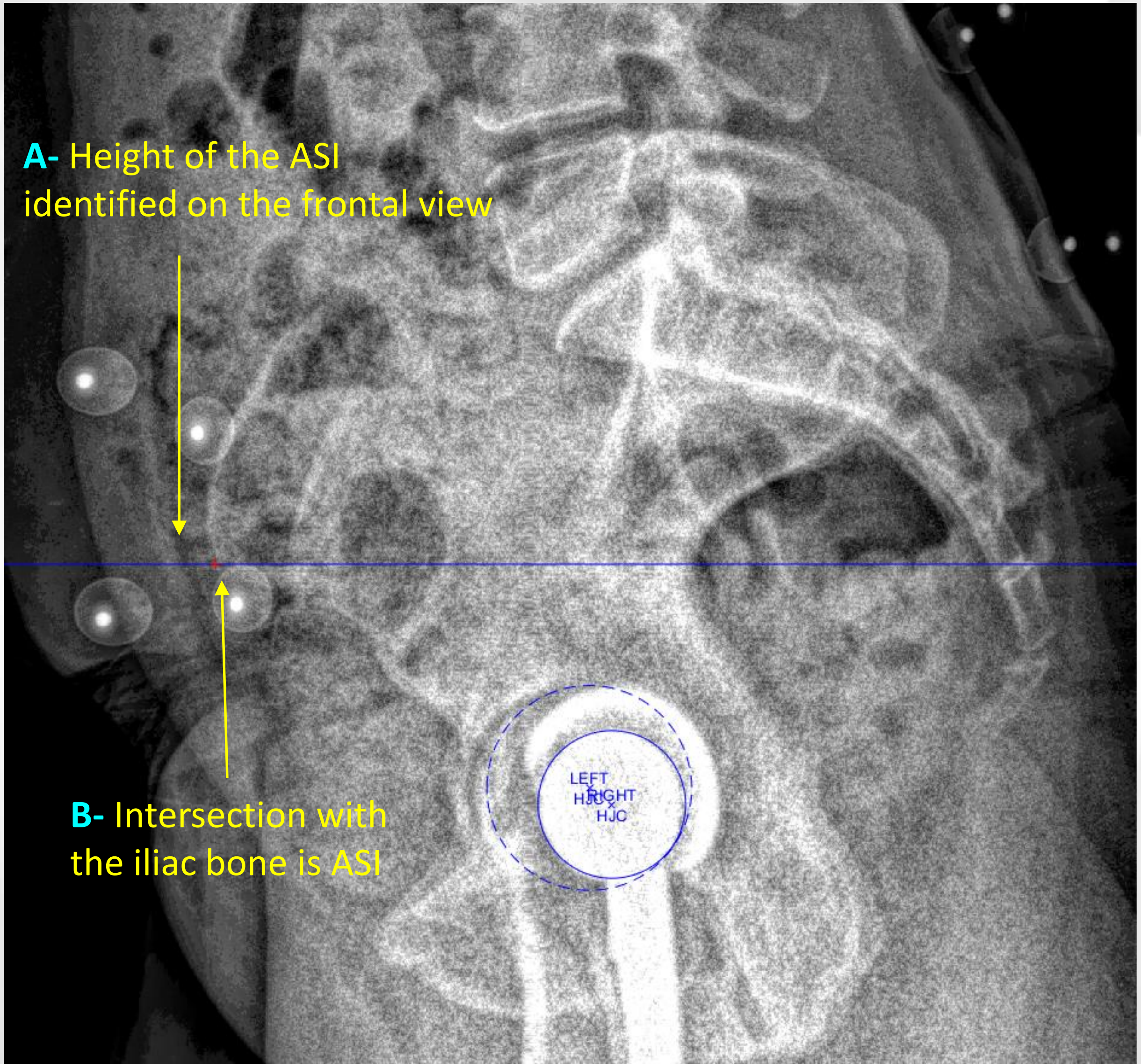

Anterior superior iliac spine

- The two methods are needed as, for some patients, the medial ridge of the superior part of the iliac bone is not visible. In this case, the second method needs to be used.

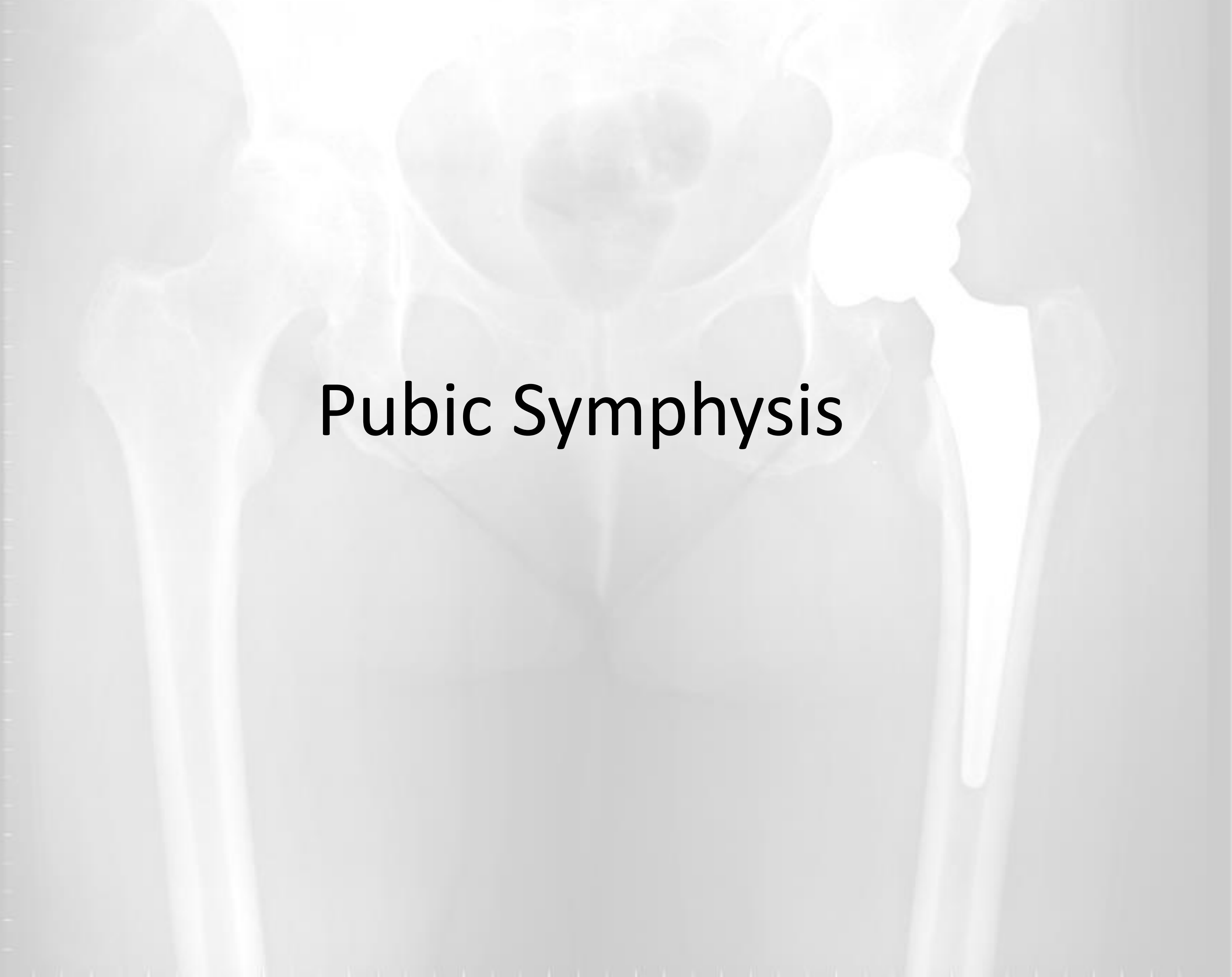

Pubic Symphysis

Supporting info to: Can the fusion of motion capture and 3D medical imaging reduce the extrinsic variability due to marker misplacements?
X. Gasparutto, J. Wegrzyk, K. Rose-Dulcina, D. Hannouche, S. Armand

Pubic Symphysis - Step 1

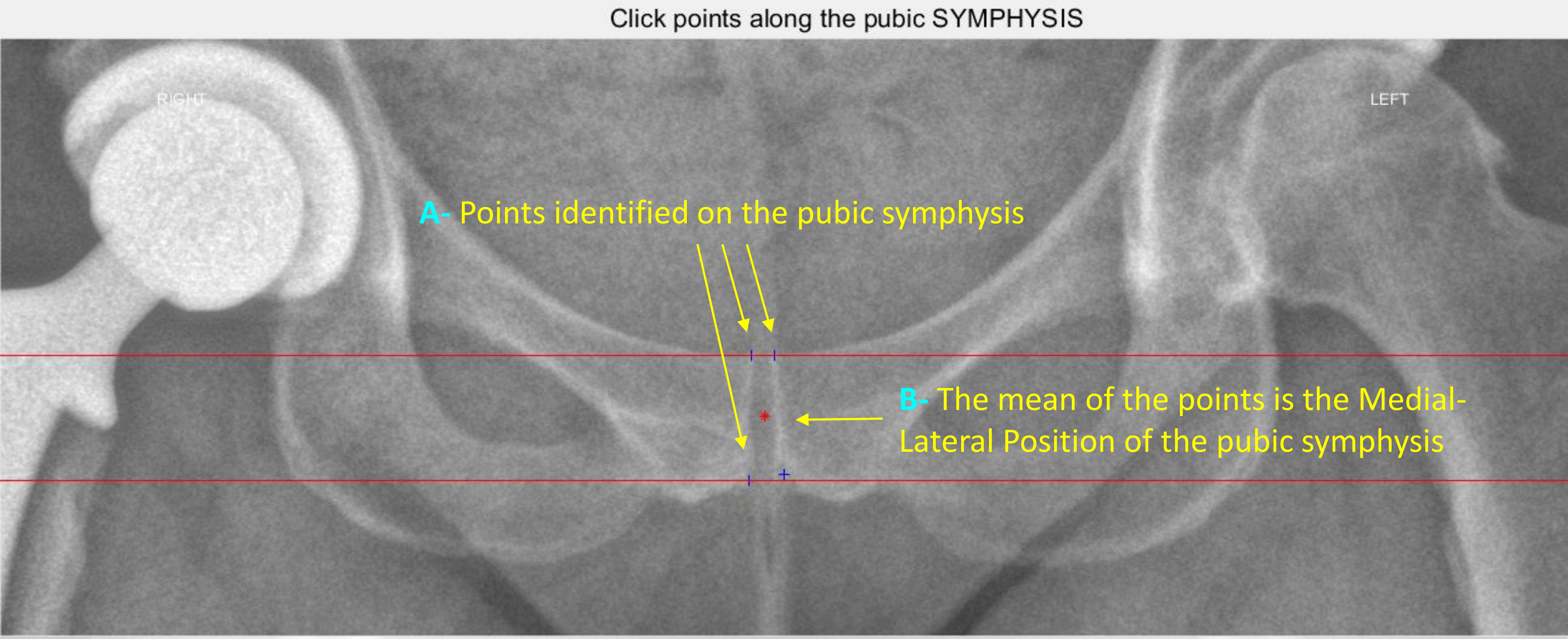

Pubic Symphysis - Step 2

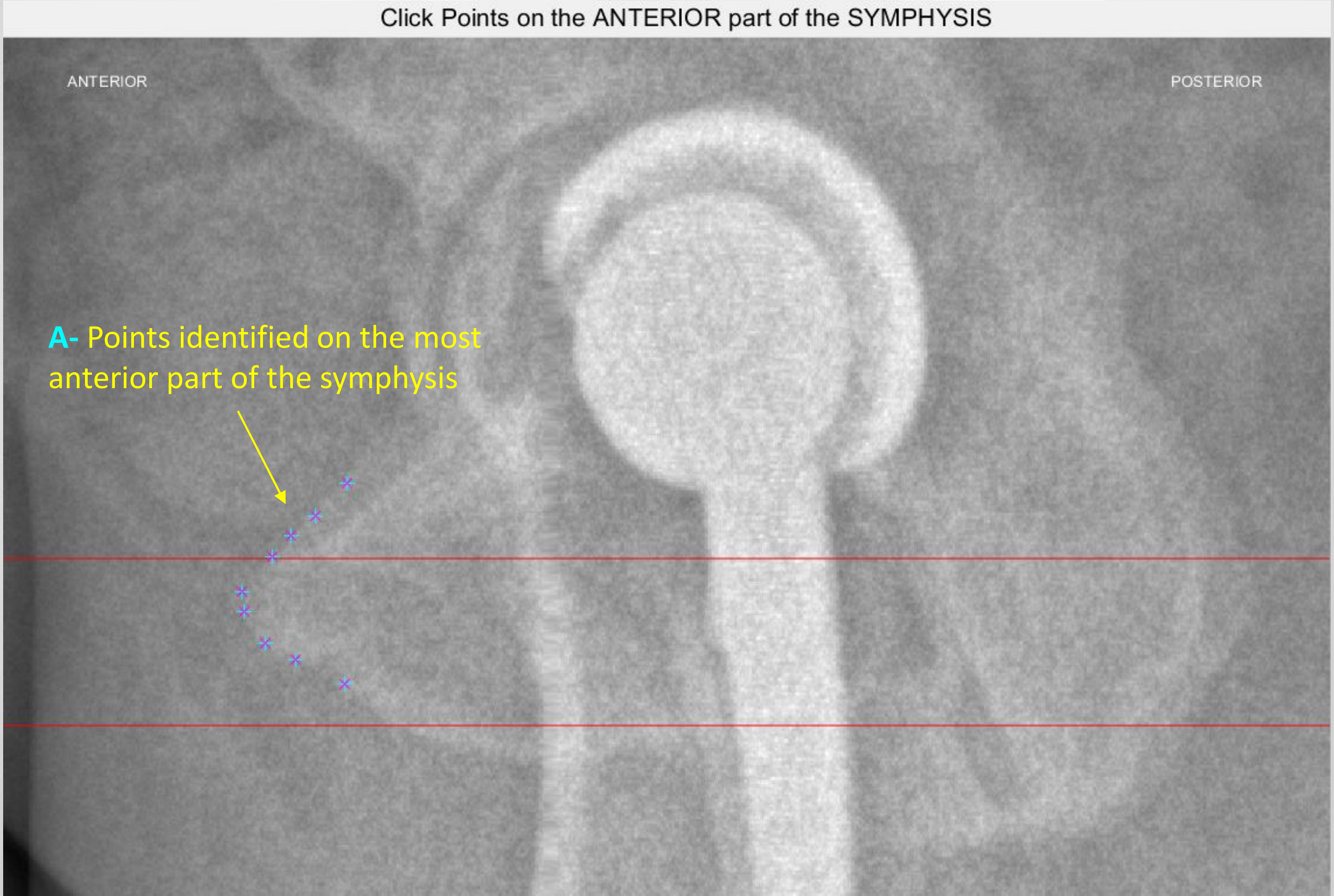

Pubic Symphysis - Step 2

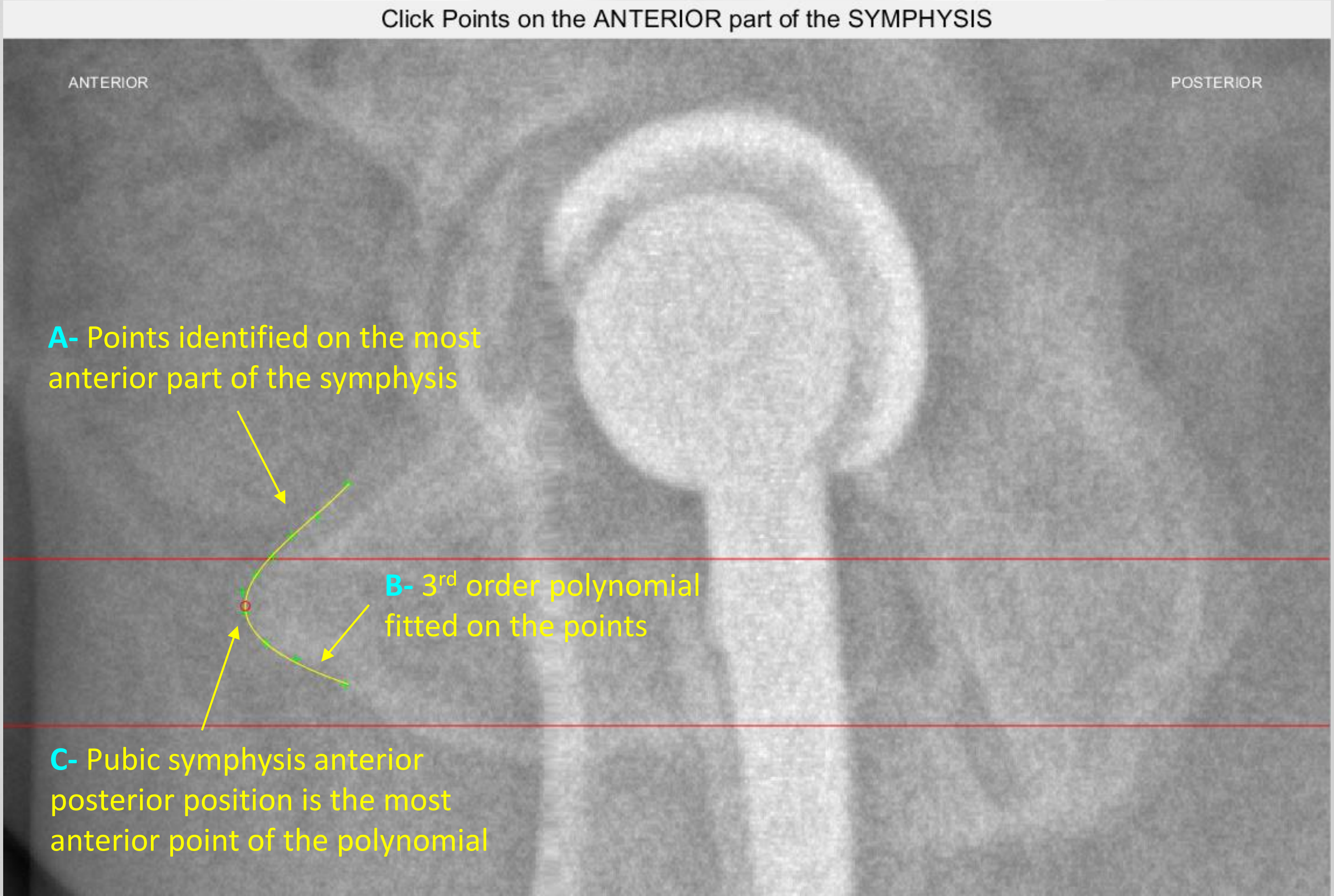

Supporting info to: Can the fusion of motion capture and 3D medical imaging reduce the extrinsic variability due to marker misplacements?
X. Gasparutto, J. Wegrzyk, K. Rose-Dulcina, D. Hannouche, S. Armand

Overview of Internal Markers And External Markers

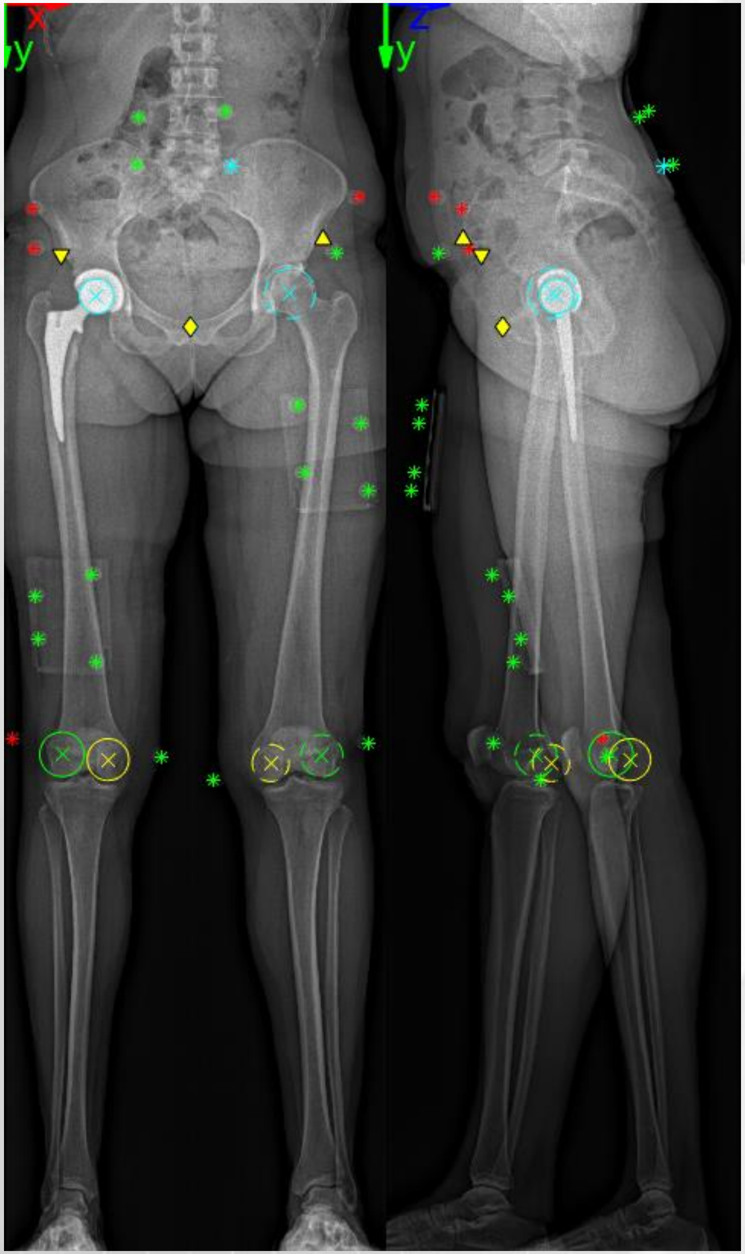

Supplement: S2 File — (PDF) [file pone.0226648.s002.pdf]
