# Supplementary material for: Can the fusion of motion capture and 3D medical imaging reduce the extrinsic variability due to marker misplacements?
Source: PLoS One. 2020 Jan 29;15(1):e0226648. doi: 10.1371/journal.pone.0226648 (PMC6988975; doi:10.1371/journal.pone.0226648)
Supplement: S3 File — (PDF) [file pone.0226648.s003.pdf]

S3 Supporting Information

Kinematics Computation

I - Definition of Local Coordinate Systems

Pelvis LCS from skin markers

Pelvis orientation from skin markers

$$\mathbf{mPSI} = (\mathbf{RPSI} + \mathbf{LPSI}) / 2$$

$$\mathbf{mASI} = (\mathbf{RASI} + \mathbf{LASI}) / 2$$

$$\begin{cases} \mathbf{y}_{PM} = (\mathbf{LASI} - \mathbf{RASI}) / \|(\mathbf{LASI} - \mathbf{RASI})\| \\ \quad \mathbf{x}_{tmp} = \mathbf{RASI} - \mathbf{mPSI} \\ \mathbf{z}_{PM} = (\mathbf{x}_{tmp} \times \mathbf{y}_{PM}) / \|(\mathbf{x}_{tmp} \times \mathbf{y}_{PM})\| \\ \mathbf{x}_{PM} = (\mathbf{y}_{PM} \times \mathbf{z}_{PM}) \end{cases}$$

$$\Rightarrow \mathbf{R}_{\mathbf{eos} \rightarrow \mathbf{PM}} = \begin{bmatrix} \mathbf{x}_{PM} & \mathbf{y}_{PM} & \mathbf{z}_{PM} \end{bmatrix}$$

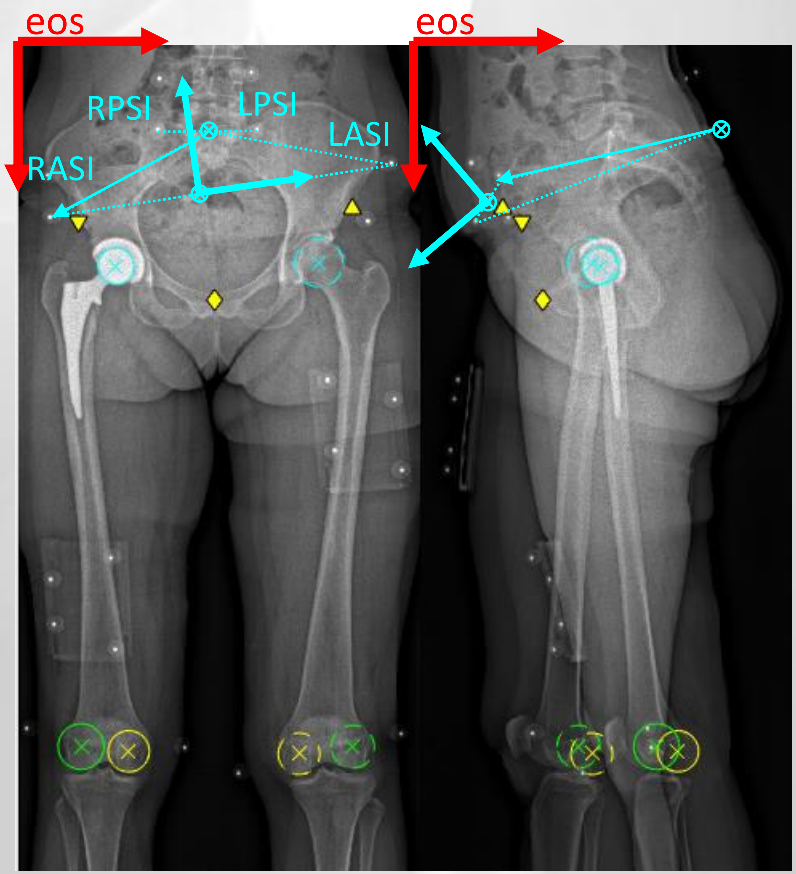

Pelvis LCS from anatomical points

Pelvis orientation from anatomical points

$$\mathbf{mASI}_a = (\mathbf{RASI}_a + \mathbf{LASI}_a)/2$$

$$\begin{cases} \mathbf{y}_{PA} = (\mathbf{LASI}_a - \mathbf{RASI}_a)/\|(\mathbf{LASI}_a - \mathbf{RASI}_a)\| \\ \quad \mathbf{z}_{tmp} = m\mathbf{ASI}_a - \mathbf{PSYM} \\ \mathbf{x}_{PA} = (\mathbf{y}_{PA} \times \mathbf{z}_{tmp})/\|(\mathbf{y}_{PA} \times \mathbf{z}_{tmp})\| \\ \mathbf{z}_{PA} = (\mathbf{x}_{PA} \times \mathbf{y}_{PA}) \end{cases}$$

$$\Rightarrow \quad \mathbf{R}_{\mathbf{eos}\rightarrow\mathbf{PA}} = \begin{bmatrix} \mathbf{x}_{PA} & \mathbf{y}_{PA} & \mathbf{z}_{PA} \end{bmatrix}$$

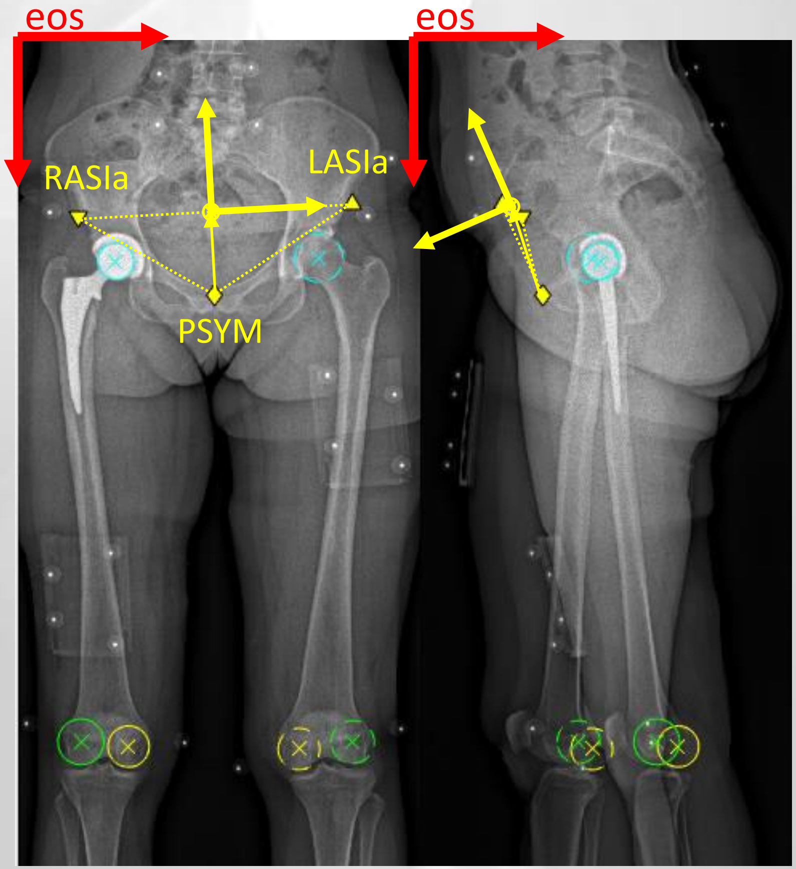

Rotation Matrix from Markers to Anat.

Orientation of Pelvis Anat. w.r.t Pelvis Mrk

$$\begin{cases} \mathbf{R}_{\mathbf{PM}\to\mathbf{PA}} = \mathbf{R}_{\mathbf{PM}\to\mathbf{eos}} . \mathbf{R}_{\mathbf{eos}\to\mathbf{PA}} \\ \mathbf{R}_{\mathbf{PM}\to\mathbf{eos}} = \left(\mathbf{R}_{\mathbf{eos}\to\mathbf{PM}}\right)^{t} \end{cases}$$

$$\Rightarrow \mathbf{R}_{\mathbf{PM}\to\mathbf{PA}} = \left(\mathbf{R}_{\mathbf{eos}\to\mathbf{PM}}\right)^{t} . \mathbf{R}_{\mathbf{eos}\to\mathbf{PA}}$$

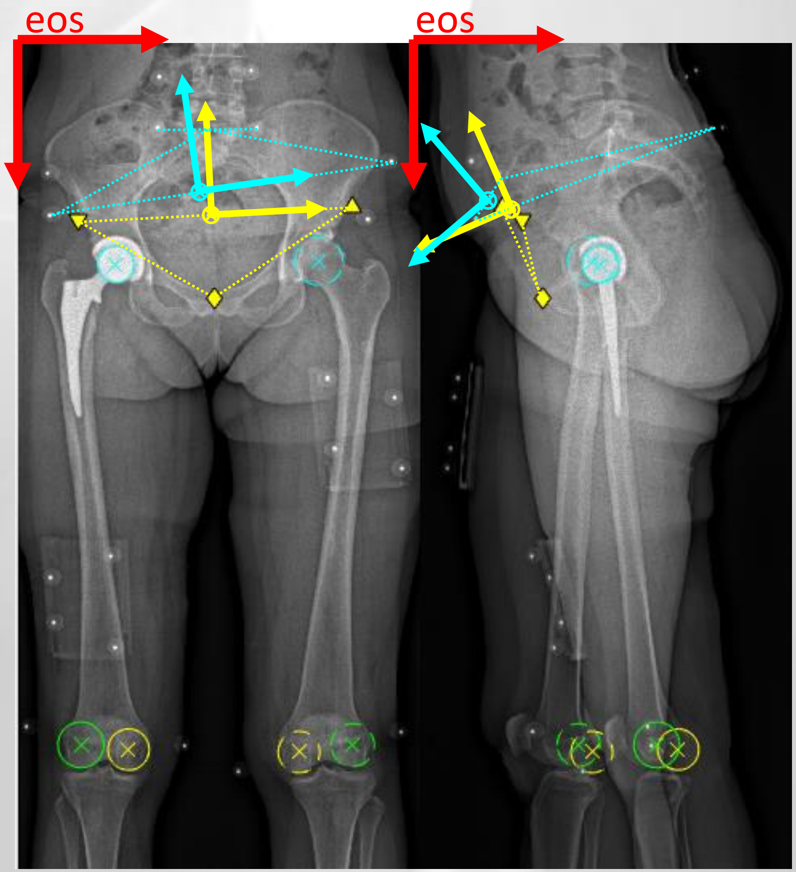

Anat. Hip Joint Center in Marker LCS

1. HJC **position** in PMLCS:

→We know position of HJCa in eos CS

→ We want the position of **HJCa** in **PMLCS**

1.1 – Change of origin:

$$\mathbf{v}\big|_{eos} = \mathbf{HJC}_a\big|_{eos} - \mathbf{mASI}\big|_{eos}$$

1.2 – Change of axis:

$$\mathbf{HJC}_a\big|_{PM} = \mathbf{R}_{\mathbf{PM}\rightarrow\mathbf{eos}}.\mathbf{v}\big|_{eos}$$

$$\mathbf{HJC}_a\big|_{PM} = \mathbf{R}_{\mathbf{PM}\rightarrow\mathbf{eos}}.\left(\mathbf{HJC}_a\big|_{eos} - \mathbf{mASI}\big|_{eos}\right)$$

In matrix form:

$$\begin{pmatrix}\mathbf{HJC}_a\\ 1\end{pmatrix}_{PM} = \underbrace{\begin{bmatrix}\mathbf{R}_{\mathbf{PM}\rightarrow\mathbf{eos}} & \mathbf{O}_{PM}\mathbf{O}_E\big|_{PM}\\ 0\quad 0\quad 0 & 1\end{bmatrix}}_{\mathbf{T}_{\mathbf{PM}\rightarrow\mathbf{eos}}}.\begin{pmatrix}\mathbf{HJC}_a\\ 1\end{pmatrix}_{eos}$$

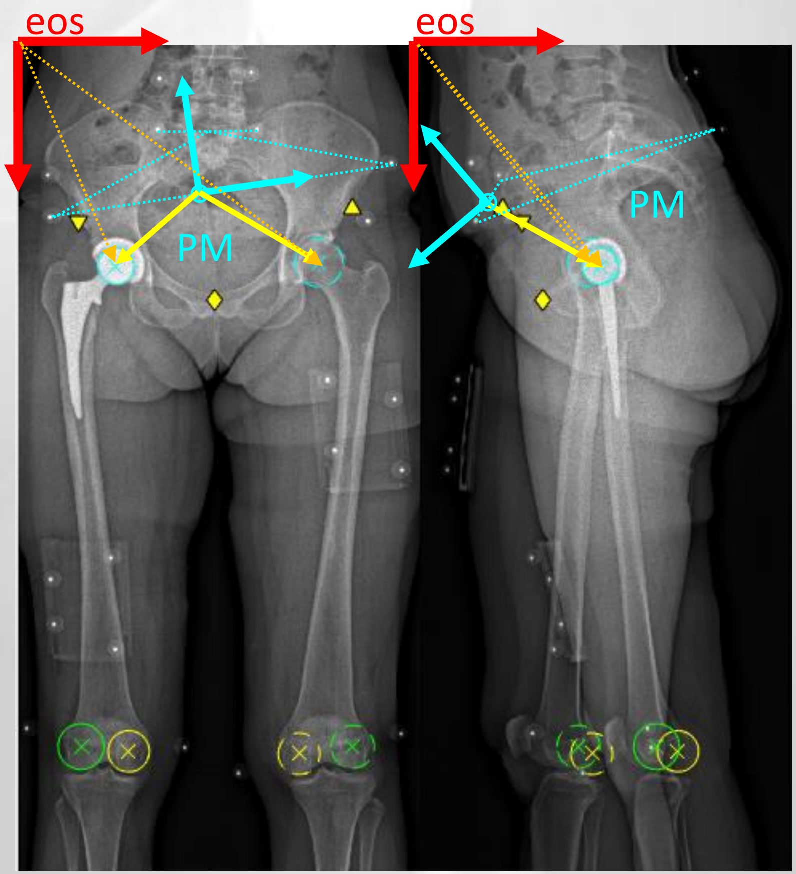

Supporting info to: Can the fusion of motion capture and 3D medical imaging reduce the extrinsic variability due to marker misplacements?
X. Gasparutto, J. Wegrzyk, K. Rose-Dulcina, D. Hannouche, S. Armand

Thigh LCS from markers (in Global not in EOS)

Orientation of Pelvis Anat. w.r.t Pelvis Mrk

1. Define plane with thigh wand, HJC, KNE
2. Define knee joint centre: point at a distance of ½ knee width & making a square triangle with HJC and KNE

$$\alpha = \arccos\left(\frac{KneeWidth/2}{\|\mathbf{HJC}-\mathbf{KNE}\|}\right)$$

3. Define LCS of the thigh based on markers

$$\begin{cases} \mathbf{z}_{TM} = (\mathbf{HJC}-\mathbf{KJC})/\|(\mathbf{HJC}-\mathbf{KJC})\| \\ \mathbf{y}_{TM} = (\mathbf{KNE}-\mathbf{KJC})/\|(\mathbf{KNE}-\mathbf{KJC})\| \\ \mathbf{x}_{TM} = (\mathbf{y}_{TM} \times \mathbf{z}_{TM}) \end{cases}$$

$$\mathbf{R}_{\mathbf{G}\rightarrow\mathbf{TM}} = \left[\mathbf{x}_{TM} \quad \mathbf{y}_{TM} \quad \mathbf{z}_{TM}\right]$$

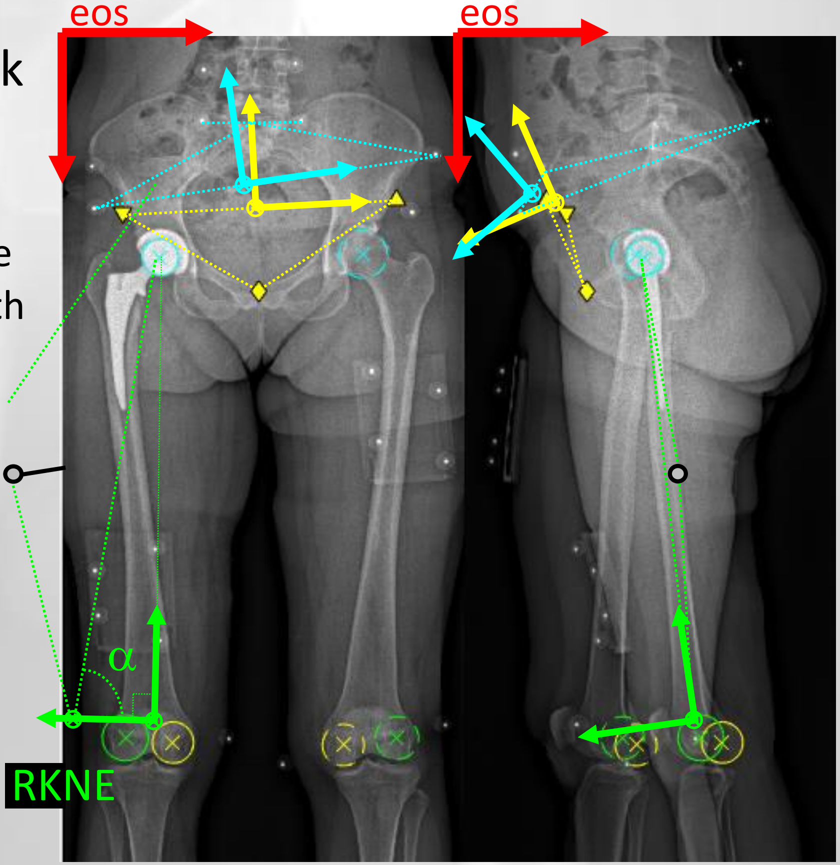

II – Computation of Pelvic & Hip Angles

Anatomical HJC during motion capture

1. Build Pelvis LCS from skin markers: $\mathbf{T}_{\mathbf{G}\rightarrow\mathbf{PM}} = \begin{bmatrix} \mathbf{R}_{\mathbf{G}\rightarrow\mathbf{PM}} & \mathbf{O}_G\mathbf{O}_{PM}\big|_G \\ 0 \quad 0 \quad 0 & 1 \end{bmatrix}$

2. Apply transformation marker to anat matrix to $\mathbf{HJC}_a\big|_{PM}$:

$$\begin{pmatrix} \mathbf{HJC}_a \\ 1 \end{pmatrix}_G = \begin{bmatrix} \mathbf{R}_{\mathbf{G}\rightarrow\mathbf{PM}} & \mathbf{O}_G\mathbf{O}_{PM}\big|_G \\ 0 \quad 0 \quad 0 & 1 \end{bmatrix} \cdot \begin{pmatrix} \mathbf{HJC}_a \\ 1 \end{pmatrix}_{PM}$$

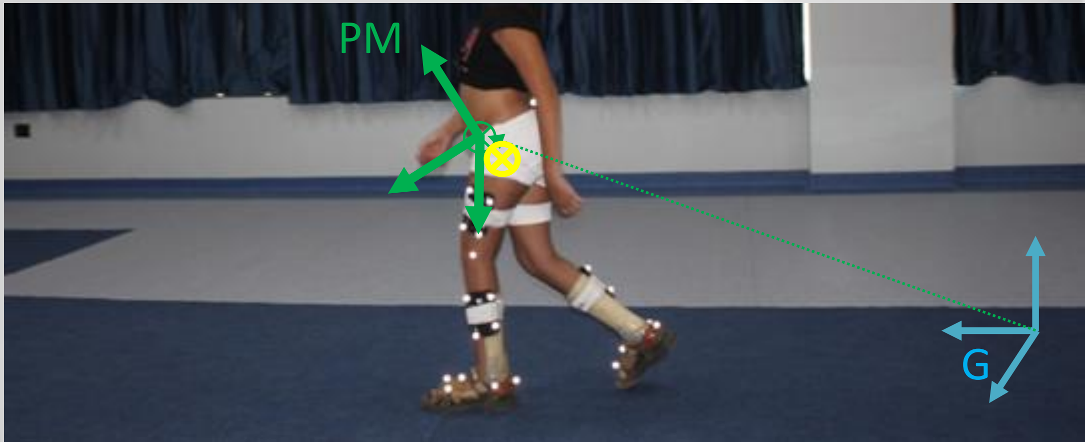

Pelvic anatomical CS during motion capture

1. Build the Pelvis LCS from skin markers: $\mathbf{R}_{\mathbf{G \rightarrow PM}}$

2. Apply rotation matrix from PM to PA

$$\mathbf{R}_{\mathbf{G \rightarrow PA}} = \mathbf{R}_{\mathbf{G \rightarrow PM}} . \mathbf{R}_{\mathbf{PM \rightarrow PA}}$$

2. Pelvis kinematics, decomposition of the matrix with a YXZ Cardanic sequence

$$\mathbf{R}_{\mathbf{G \rightarrow PA}} = \mathbf{R}_{\mathbf{YXZ}}$$

$\rightarrow$ **Pelvic angles**

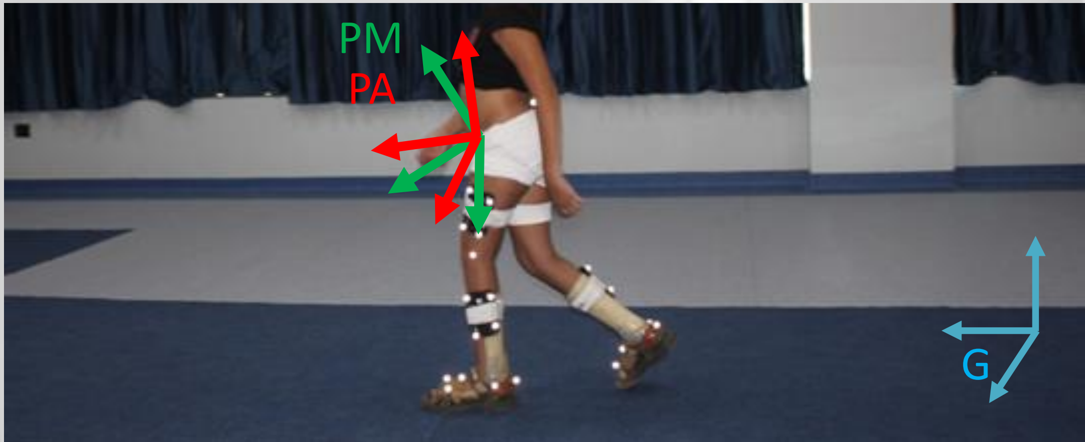

Hip Kinematics

1. Build the Thigh LCS from skin markers: $\boxed{\mathbf{R}_{\mathbf{G}\to\mathbf{TM}}}$
2. Build rotation matrix $\mathbf{R}_{Z\pi}$ that applies a rotation of 180 degrees around the Z axis. It is used to match the signs conventions of the joint angles.
3. Build rotation matrix of the right and left hip joints

Right Hip: $\mathbf{R}^{r}_{\mathbf{P.}\to\mathbf{TM}} = \left(\mathbf{R}_{\mathbf{G}\to\mathbf{P}}.\mathbf{R}_{Z\pi}\right)^{-1}.\mathbf{R}^{r}_{\mathbf{G}\to\mathbf{TM}}$

Left Hip: $\mathbf{R}^{l}_{\mathbf{P.}\to\mathbf{TM}} = \left(\mathbf{R}_{\mathbf{G}\to\mathbf{P}}.\mathbf{R}_{Z\pi}\right)^{-1}.\mathbf{R}^{l}_{\mathbf{G}\to\mathbf{TM}}.\mathbf{R}_{Z\pi}$

4. Decomposition of the matrices with a **YXZ** Cardanic sequence

$$\mathbf{R}^{\cdot}_{\mathbf{P.}\to\mathbf{TM}} = \mathbf{R}_{\mathbf{YXZ}}$$

$\rightarrow$ **Hip angles**

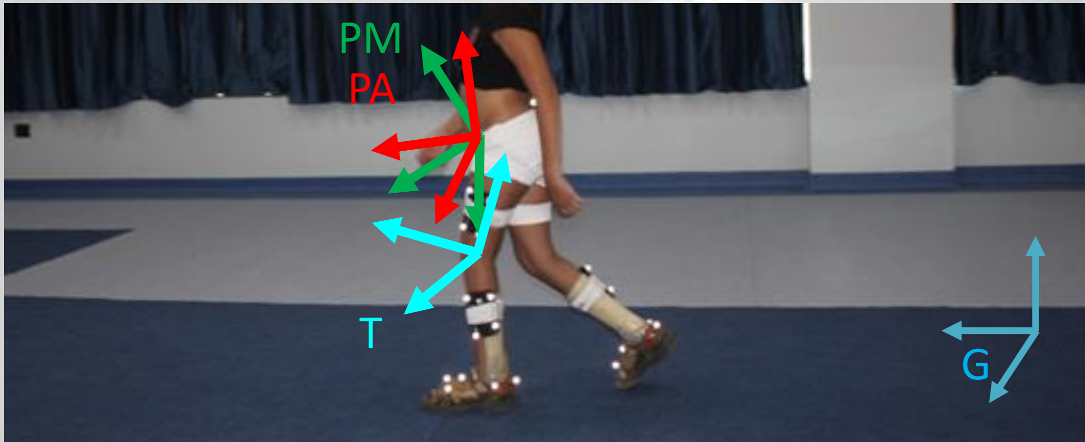

Supporting info to: Can the fusion of motion capture and 3D medical imaging reduce the extrinsic variability due to marker misplacements?
X. Gasparutto, J. Wegrzyk, K. Rose-Dulcina, D. Hannouche, S. Armand

Rotation Matrix Ryxz

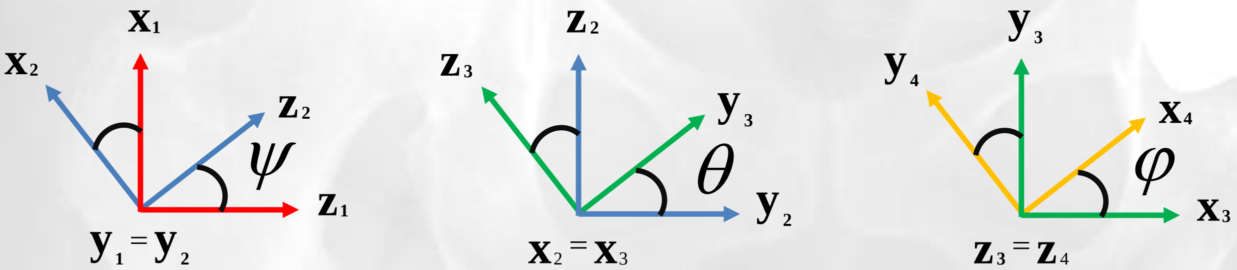

$$R_{YXZ} = \begin{bmatrix} c\psi.c\varphi + s\psi.s\theta.s\varphi & -c\psi.s\varphi + s\psi.s\theta.c\varphi & s\psi.c\theta \\ s\varphi.c\theta & c\varphi.c\theta & -s\theta \\ -s\psi.c\varphi + c\psi.s\theta.s\varphi & s\psi.s\varphi + c\psi.s\theta.c\varphi & c\psi.c\theta \end{bmatrix}$$

Pelvis Angles

Angle	Rot. Axis	Angle
Ψ	**yG**	Tilt
θ	**yG** x **zP**	Obliquity
φ	**zP**	Rotation

Hip Joint Angles

Angle	Rot. Axis	Angle
Ψ	**yG**	Tilt
θ	**yG** x **zP**	Obliquity
φ	**zP**	Rotation
