# Supplementary figures and images for: Can the fusion of motion capture and 3D medical imaging reduce the extrinsic variability due to marker misplacements?
Source: PLoS One. 2020 Jan 29;15(1):e0226648. doi: 10.1371/journal.pone.0226648 (PMC6988975; doi:10.1371/journal.pone.0226648)

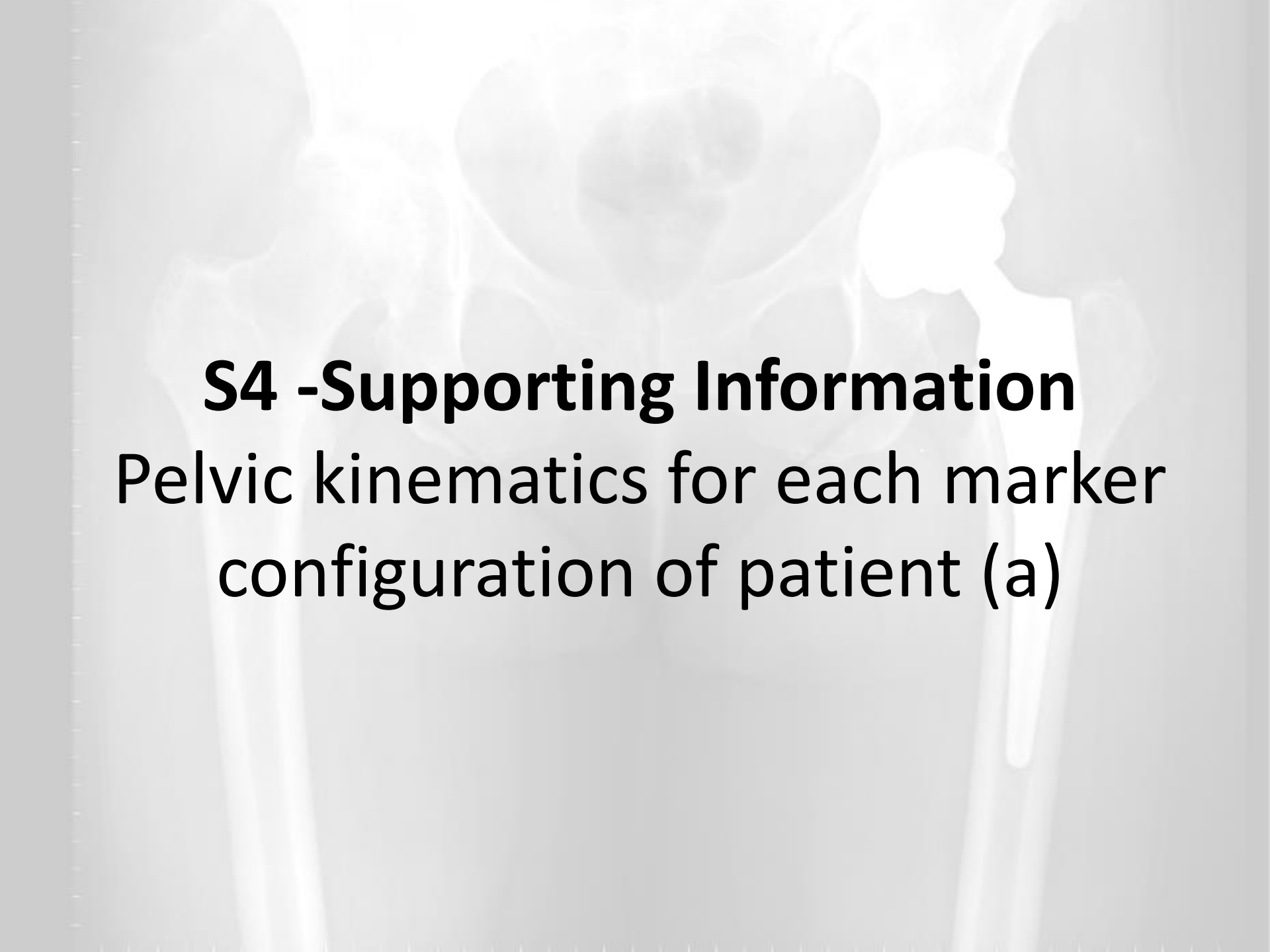

# **S4 -Supporting Information**

## **Pelvic kinematics for each marker configuration of patient (a)**

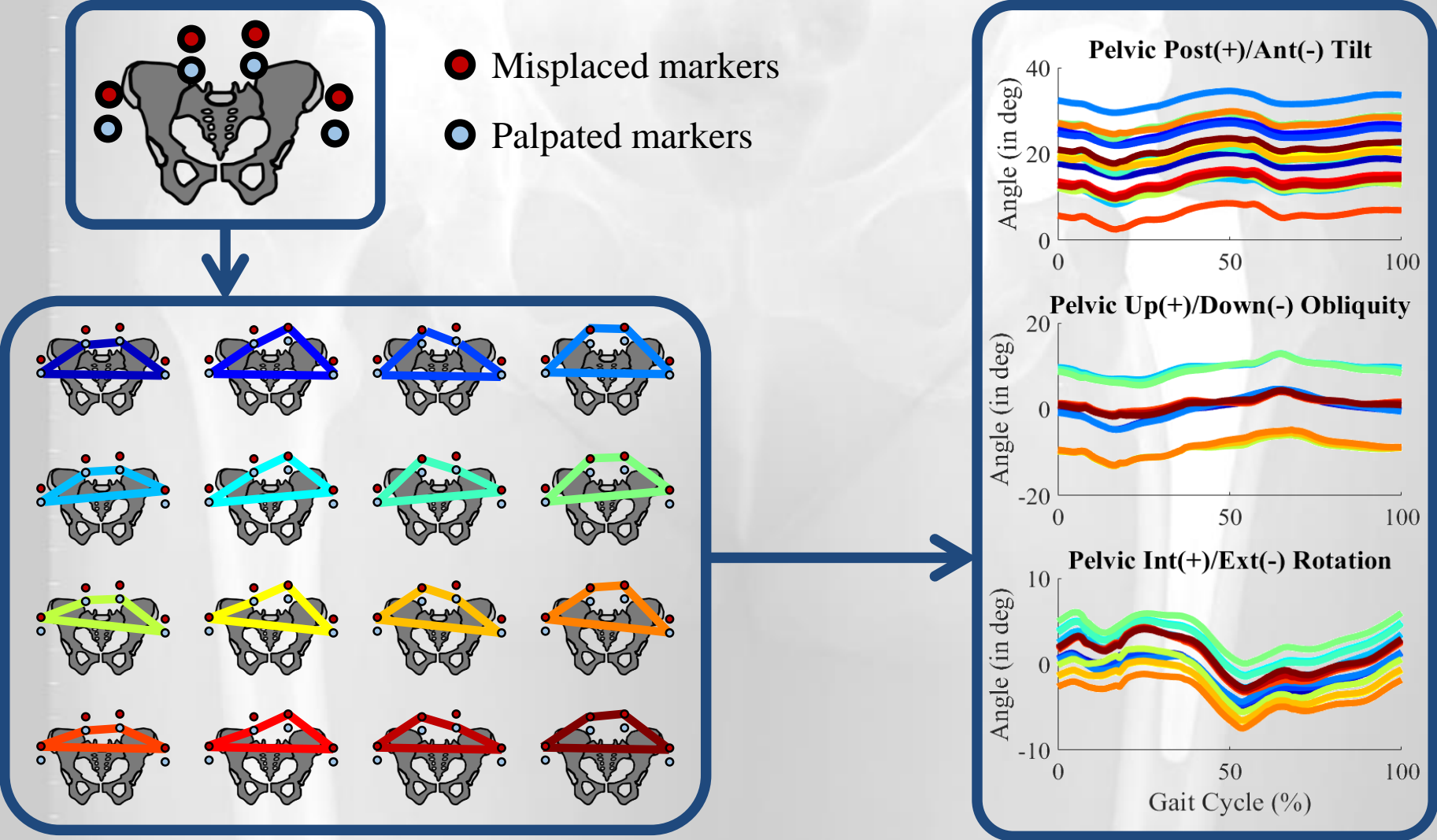

Supplement: S4 File — (PDF) [file pone.0226648.s004.pdf]
