# Supplementary material for: Can the fusion of motion capture and 3D medical imaging reduce the extrinsic variability due to marker misplacements?
Source: PLoS One. 2020 Jan 29;15(1):e0226648. doi: 10.1371/journal.pone.0226648 (PMC6988975; doi:10.1371/journal.pone.0226648)
Supplement: Attachment — Submitted filename: Response to Reviewers_v2.docx [file pone.0226648.s005.docx]

Dear Dr Zhang,

We would like to thank you and the reviewers for your time and for the constructive feedback that helped improve the quality of the paper. Thank you also for sharing references with us, we added Zheng et al. (2014) as a reference to complete the introduction of the paper. We will share our data on Yareta and our codes on GitLab. Only one part of the code is currently shared, we are currently preparing the rest of the code and data used in the paper.

Please find the answer to the reviewers in red in the text below.

**Review Comments to the Author**

**Reviewer #1:** In the submitted manuscript, the authors present an investigation into the feasibility of fusing optical motion capture with bi-plane X-rays to reduce kinematic variability due to misplaced skin markers (vertical offset, axial rotation, anteversion, and obliquity). In this study, they specifically focused on pelvis and hip kinematics during a preferred-speed walking task completed by four subjects. Hip joint centres (HJCs) were estimated by either regression with the optical motion capture data or manually by the bi-plane X-rays. Pelvis kinematics were estimated using a pelvic anatomical frame defined by either the external markers for optical motion capture (standard) or the internal markers provided by the bi-plane X-rays (fusion). Hip kinematics were estimated using standard pelvis kinematics + regression-based HJC, fusion pelvis kinematics + regression-based HJC, and fusion pelvis kinematics + X-ray-based HJC. Nonparametric statistical analyses were conducted for comparing the different pelvis and hip kinematics. The supporting information files were excellent and clear.

Thank you for those comments.

The main weakness of this paper is missing or unclear information. For example, it was not clear until the results that each patient effectively represents a different kind of marker misplacement, meaning the markers for patient (a) were given a vertical offset, the markers for patient (b) were given an axial rotation, and so on.

We added a sentence to the Material & Method section to clarify this point:

L.91 “Each patient had one configuration of marker misplacement”

Next, the authors offer no explanation for why they are conducting nonparametric statistical analyses (I assume it is because the data are non-normally distributed, in which case they should state as much).

The number of patients was too low to use parametric statistical analysis. The following sentence was added to the method section:

L.187: “Non-parametric statistical analysis was performed due to the low number of patients included in the study.”

Also, the figure captions for Figs. 3, 4, and 6 need to be more descriptive. For example, in Fig. 3, what do the dashed lines around the hip joint centres identified by the X-ray mean, why are there 12 results for the HJC regression for each hip, specify again that each subfigure is a different patient, etc.

A sphere fitting of the femoral head was performed on the bi-plane x-ray to identify the hip joint centres. The dashed lines represent this sphere projected on both planes, i.e. the dimension of the femoral head. For each HJC obtained with regression, there are actually 16 points (one for each combination of markers) but the points overlap.

We added the following sentence to the description of Figure 3:

“Each figure represent one patient with a different marker misplacement. The points represent the hip joint center location and the dashed circles represent the size of the femoral head identified by sphere fitting.”

Also, in reference to Figs. 4 and 6, could the authors provide some insight in the main body of the paper as to why some subplots appear to have discrete bands of results (see, for example, the plot for Pelvic Post(+)/Ant(-) Tilt for Anteversion Misplacement)?

All subplots have discrete bands of results but some are more striking than others (as for your example). The misplacement error introduced in the study was discrete and rather large (4cm) which leads to this discrete pattern. The way curves are grouped depend on the marker configurations. As an example, for the pelvic obliquity and pelvic rotation, each group is associated with a combination of the ASI markers (either 2 palpated, 2 misplaced or one of each).

For your example (Pelvic Post(+)/Ant(-) Tilt for Anteversion Misplacement) there is 5 different groups also associated to a specific combination of markers that we describe below (from highest tilt to lowest tilt)

- Group 1: all markers are the misplaced ones (1 curve) – maximal tilt
- Group 2: all markers but one are misplaced (4 curves)
- Group 3: 2 markers misplaced and 2 palpated markers (6 curves)
- Group 4: all markers but one are palpated (4 curves)
- Group 5: all markers are palpated (1 curve) – minimal tilt

However, the groups seen on the tilt for the other misplacement are different, those groups depend on the misplacements. To summarize, some combinations of skin markers maximize the values on one angle, others minimize it. In between, there are multiple combinations that lead to similar values of joint angles. As the misplacement was discrete, the results shows discrete curves. We added a figure in Supplementary Material to clarify this issue. It depicts every marker combination with a different colour and the associated pelvic angles are plotted with the same colour and make it easier to understand what we did.

If you find it appropriate it could be added to the main body of the paper but it might be too much figures.

Finally, the sentence on Lines 189-190 feels likely out of place. It would be better situated in the “Bi-plane X-Rays” section starting on Line 98.

This sentence was moved to the paragraph “Bi-plane X-rays” in the method section.

This study was well-designed and the paper was fairly well-written. As such, it is the opinion of this reviewer that it should be accepted with minor revisions. However, the authors are encouraged to carefully proofread the paper when addressing the previous comments (for example, I believe R_L->PM on Line 141 should be R_L->PE and “wish” on line 322 is missing the “w”).

R_L->PM was changed to R_L->PE and the missing “w” was corrected.

**Reviewer #2:** Reducing the extrinsic vrability occurred from marker misplacement is an essential issue for current motion studies, which is still lay hopes on an effective method. Many researches attempted analyzing the motion data with assisted medical imaging technology, but the effectiveness is weak. This work made a progress of solving this problem by using a fusion way of capturing motion and bi-plane X-ray imaging. This could capture the bone motion rather than the traditional sufrace motion. The work is technically sound. However, using bi-plane X-ray imaging is not that innovative, and no modelling was introduced in the work.

Thank you for those comments. We do not claim to introduce a novel method. As underlined in the introduction (L70-72), the point of the paper is to demonstrate that the fusion of motion capture and medical imaging could help reducing the variability due to marker misplacement. This could work with a different medical imaging method, as suggested in the discussion (L299 - 300). No specific modelling was required for this study.

The illustration are unclear with blurred figures.

This is due to the pdf generation of PlosONE, there is a link on the top right of the pdf to download the figures with a higher quality.

The literature reviews and their references are not up to date.

We added the following references to the papers’ introduction:

- Akpinar B, Thorhauer E, Tashman S, et al. Tibiofemoral Cartilage Contact Differences Between Level Walking and Downhill Running. Orthop J Sport Med 2019; 7: 1–7.
- D’Isidoro F. How does the hip joint move? Techniques and applications. ETH Zurich, 2018.
- Zheng L, Li K, Shetye S, et al. Integrating dynamic stereo-radiography and surface-based motion data for subject-specific musculoskeletal dynamic modeling. J Biomech 2014; 47: 3217–3221.
- Valente G, Crimi G, Vanella N, et al. nmsBuilder: Freeware to create subject-specific musculoskeletal models for OpenSim. Comput Methods Programs Biomed 2017; 152: 85—92.
